# The Basics of Evolution Strategies: The Implementation of the Biomimetic Optimization Method in Educational Modules

**DOI:** 10.3390/biomimetics9070439

**Published:** 2024-07-18

**Authors:** Olga Speck, Thomas Speck, Sabine Baur, Michael Herdy

**Affiliations:** 1 Cluster of Excellence livMatS @ FIT—Freiburg Center for Interactive Materials and Bioinspired Technologies, University of Freiburg, Georges-Köhler-Allee 105, 79110 Freiburg, Germany; thomas.speck@biologie.uni-freiburg.de; 2 Plant Biomechanics Group @ Botanic Garden Freiburg, University of Freiburg, Schänzlestr. 1, 79104 Freiburg, Germany; 3 Ingenieurbüro Herdy (IBH), Kaiserdamm 4, 14057 Berlin, Germany

**Keywords:** biological evolution, biomimetic optimization, brachistochrone curve, fitness, mutation, recombination, selection

## Abstract

With a focus on education and teaching, we provide general background information on bioinspired optimization methods by comparing the concept of optimization and the search for an optimum in engineering and biology. We introduce both the principles of Darwinian evolution and the basic evolutionary optimization procedure of evolution strategies. We provide three educational modules in work sheets that can be used by teachers and students to improve their understanding of evolution strategies. The educational module “Optimization of a Milk Carton” shows that the material consumption in producing a milk carton can be minimized using an evolution strategy with a mutative step size control. The use of a standard dice and a pocket calculator enables new milk cartons to be generated, with the offspring having the lowest material consumption becoming the parent of the next generation. The other educational modules deal with the so-called brachistochrone problem. The module “Fastest and Shortest Marble Track” provides a construction plan for a marble track whereby students can experimentally compare the “path of shortest length” with the “path of shortest time”. The EvoBrach software, is used in the module “Various Marble Track Shapes” to compare the running times of a marble on a straight line, a parabola, and a brachistochrone. In conclusion, the introduction to the biomimetic method of evolution strategies and the educational modules should deepen the understanding of both optimization problems and biological evolution.

## 1. Introduction

Humankind has always striven to improve objects or processes and to find the best solution to given problems, i.e., to achieve the optimal solution. An entire sub-discipline of mathematics is even devoted to the development of algorithms for solving various optimization problems. In addition, nature-inspired meta-heuristics (i.e., higher level procedures or heuristics) such as Evolutionary Algorithms (EAs), Ant Colony Optimization, and Particle Swarm Optimization have been developed to find a sufficiently good solution to an optimization problem [1,2,3]. These days, there are attempts to build important bridges between the two rather separate communities of researchers and practitioners in mathematical programming and nature-inspired meta-heuristics [4,5].

With the increasing availability and power of computers in the mid-1960s and early 1970s, several optimization methods based on the optimization principles of Darwinian evolution were developed almost simultaneously, but independently [6,7,8]. The nature-inspired optimization methods include evolution strategies (ESs) [9,10], Genetic Algorithms (GAs) [11], and Evolutionary Programming (EP) [12]. The GA emphasizes genetic mechanisms at the level of chromosomal abstraction. ESs, on the other hand, represent evolutionary events at the phenotypic level. Finally, EP considers evolution at the level of entire populations or species [13]. These individual models were long used in competition with each other. Individual representatives of an optimization strategy have tried to prove theoretically and empirically the superiority of their preferred approach over other approaches [8]. However, the superiority of one model over the others is impossible to establish since each strategy has its own strengths and limitations [13].

Today, they are regarded as sub-classes of EAs and are often used in combination with each other [8,13]. The basic philosophy of these models is largely the same. The idea is always to subject a collection of solutions, a so-called population, to simulated biological evolution. New solutions are generated by randomly changing the parameters of individuals (cf. biological mutation, i.e., small random changes in genetic information) and by recombining the parameter values of different candidates (cf. biological recombination, i.e., genetic reshuffling), which are then tested for their efficiency. Analogous to biological selection (i.e., differential survival and reproduction of individuals), the less efficient solutions are eventually weeded out, leaving only the solutions that fit best. These then form the basis for further evolutionary cycles [14]. Although all EAs are inspired by the principles of evolution such as mutation, recombination, selection, and isolation (cf. Section 2), they are implemented differently. Whereas recombination plays an important role in the GAs, mutations play the central role in the ESs and EP, whereby, in EP, normally distributed random numbers are selected for the mutation step sizes (cf. Section 3.5 and Section 3.6) [7].

Recently, evolutionary computation has shown a gap in the consideration of epigenetic inheritance, a key building block in modern interpretations of biological evolution. In biology, epigenetics is the study of heritable traits or stable changes in cell function that are not caused by changes in the DNA sequence, but by the regulation of gene expression. Such epigenetic effects result from environmental factors. Yuen et al. [15] introduced several ways to include epigenetic mechanisms in the EA, allowing the transfer of epigenetic information to future generations.

In this article, we focus on ESs that were developed by Ingo Rechenberg and Hans-Paul Schwefel in the mid-1960s and early 1970s [9,10]. ESs abstract the basic principles of biological evolution from their natural context in order to apply the evolutionary loop for engineering optimization problems [7]. With the help of ESs, difficult optimization problems can be solved for which standard optimization methods cannot be applied. The reason may be that the prerequisites are absent, for example only noisy evaluations or no mathematical formulations of the target function are available [6]. The main field of application of ES is numerical optimization, i.e., the minimization or maximization of a function [7]. However, combinatorial tasks can also be optimized highly successfully by ESs [16,17,18].

### 1.1. Concept of Optimization

The term “optimum” is derived from the Latin optimus, which can be translated as “best, most excellent” and in technology means “highest achievable measure” or “highest achievable value” [19] with regard to a target to be achieved under certain conditions. Accordingly, the term “optimization” refers to a process, i.e., the search for such a maximum under given conditions and objectives. However, the search for the highest achievable value only makes sense if several values are available allowing comparisons. If a problem can only be solved in one way, no alternative can be considered, and therefore, nothing can be optimized. The same is true for a problem for which uncertainty exists as to whether it can be solved at all. The latter situation requires an invention or discovery rather than optimization [14,20]. Only when two or more solutions exist for a problem, and when these fulfill the optimization criterion differently well under the same conditions, it is an optimization problem in the true sense [10].

**Figure 1:**
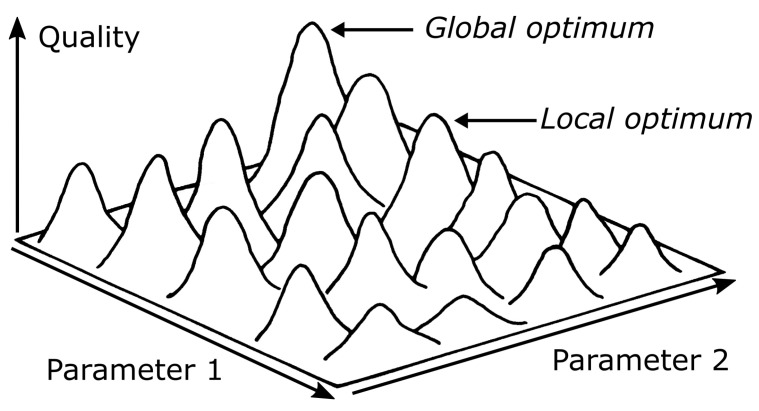
Three-dimensional quality landscape of a maximization problem with two parameters. The landscape exhibits one global maximum and several local maxima.

Usually, the variable to be optimized is influenced by several factors, some of which are fixed from the beginning, namely the constants. However, the variables or parameters are of particular importance because they can influence the quality of a potential solution. An optimization problem in which the variable to be optimized depends on two parameters can be illustrated graphically in a particularly impressive way. If the two parameters are plotted horizontally, and if the corresponding value of the variable to be optimized, namely the quality, is plotted vertically, the result is a three-dimensional quality landscape (Figure 1). Each parameter combination symbolizes a proposed solution. All parameter combinations taken together form the so-called search space in which the parameter combination with the best quality value is sought. Depending on whether this is a maximization or minimization problem, we wish to find either the highest peak or the deepest valley in the quality landscape range. In the case of a minimization problem, the search for the optimum can be understood as the search for the deepest valley. In the case of a maximization problem, it can be understood as the search for the highest peak in the quality landscape. Of course, it is always desirable to find the global optimum. However, in practice, it often makes more sense to accept a local optimum that is only slightly worse than that achieved after the investment of even more time and money in further optimization [8].

Generally, the objective function of a minimization problem can be described by Equation (1) and a maximization problem by Equation (2) [6]:

(1) f(x)→min!

(2) f(x)→max!

Whereas the set of all theoretically possible solutions is given by the search space, constraints (i.e., restrictions), which can be described in the form of equations or inequalities, determine the practically relevant solution space [14,20]. Thus, optimization problems include not only an objective function, but also constraints that must be satisfied by any solution. A constrained optimization problem can generally be described by Equations (3) and (4), where *h* and *g* are the parameters [6]:

(3) $$h_{i}\left(x\right)\leq 0,i=1,\ldots ,N_{h}$$

(4) $$g_{i}\left(x\right)\leq 0,i=1,\ldots ,N_{g}$$

There are several types of constraints. For example, the parameters can take only certain discrete values or lie within predefined intervals. In addition, only certain parameter combinations are allowed or the objective function can only be calculated for certain parameter combinations [6]. Examples of objective functions and various constraints are described in the three educational modules presented in Section 5.

### 1.2. Aim of the Project

Here, we provide general background information on EAs (Section 1), we compare the concept of optimization and the search for an optimum in engineering and biology (Section 1.1), and we present both the principles of Darwinian evolution (Section 2) and the basic evolutionary optimization procedure of ESs (Section 3). In Section 4, we introduce several real-life applications of ESs to engineering. In addition, we present three educational modules with background information (Section 5) and work sheets (Supplementary Materials Files S1–S4), which can be used by lecturers, teachers, and students from high schools, colleges, and universities to attain a better understanding of ESs. We provide the educational modules “Optimization of a Milk Carton”, “Fastest and Shortest Marble Track”, and “Various Marble Track Shapes” together with the freely available EvoBrach software (version August 2013).

## 2. Biological Evolution

In biology, optimization occurs through biological evolution, which has been shown to create structures that are highly adapted to their environment, i.e., optimized with respect to their environmental requirements. In the plant kingdom, we distinguish between response, acclimation, and adaptation as a reaction to environmental changes [21], whereby adaptation is the inspiration for ESs. Individual plants can respond reversibly within seconds to minutes by reconfiguration of their plant organs. A prime model of response is the reversible streamlining of plants and their organs under wind loads. Moreover, individual plants can acclimate within days to months through changes in gene expression. These “trained” plants exhibit non-hereditary alterations of growth patterns, such as the reduction in shoot elongation caused by mechanical stimulation. Adaptation, however, takes place within populations of plants over an evolutionary time. Because of changes in their genetic information, “adapted” plants exhibit a variety of hereditary traits [21,22]. Examples are fire-adapted plants that form thick bark layers that protects the living tissue inside the trunk. These so-called pyrophytes benefit from fire directly by means of fire-initiated cone opening and seed release, and indirectly because fewer competing plants of fire-sensitive species remain and because their seeds can germinate in the ash-fertilized soil [23,24].

The ES is a biomimetic optimization method, abstracted from the principles of Darwinian evolution [7,16,25]. Evolution only works because living beings constantly reproduce, thereby producing more offspring than can survive because of limited resources (e.g., food, habitat, reproductive partners). The individual members of a species, which differ from each other through the mutation and recombination of parental genes, are, therefore, inevitably in competition with each other. Those individuals in a population that are best adapted to their environment have a greater chance of surviving and ultimately reproducing than their less well adapted competitors [6,20]. Differential reproductive and survival success represent the evolutionary principle of natural selection [25]. The ES utilizes the variation of genetic information (genotype) through mutation and recombination, and selection based on the phenotype in an iterative evolutionary loop analogous to the variation and selection experienced by the biological parent and offspring generations, which in turn represent the next parent generation, and so on. Table 1 gives an overview of the comparison between biological evolution and the ES. The biological meanings of mutation, recombination, selection, and isolation are explained below.

### 2.1. Mutation

The term “mutation” is derived from the Latin *mutatio*, which can be translated as “change”, “alteration”, or “transfer” [26]. In biology, mutations are small random changes in genetic information. They can occur spontaneously (e.g., during the duplication of genetic information) or be induced by mutagens such as radiation or chemicals. Only changes in the genetic material of cells involved in the production of offspring are of evolutionary significance. These germline mutations can be either beneficial or detrimental to the individual. Only comparatively rare beneficial mutations result in evolutionary development [20,26].

### 2.2. Recombination

The term “recombination” is derived from the Latin *re*, which can be translated as “again”, and *combinatio*, which means “union” [27,28]. In biology, genetic recombination, also known as genetic reshuffling, refers to the new combination of paternal and maternal genetic material in the context of sexual reproduction. Recombination occurs during meiosis between the genetic material of the generative cells and can result in cells simultaneously containing some original paternal or maternal chromosomes and some chromosomes that have recombined. The process of fertilization, thus, ultimately results in offspring that, according to the law of chance, have newly combined hereditary traits compared with those of their ancestors. In diploid organisms, the effect of the newly distributed genetic material on the expression of traits can be either intermediate (i.e., the expression of traits is influenced equally by both alleles of a gene) or dominant–recessive (i.e., the effect of the dominant allele masks the effect of the recessive allele) [20,29].

### 2.3. Selection

Selection (from the Latin *selectio*, translated as “choice” or “selection” [30]) is considered to be the real controlling element of evolution because it compensates for the initial “randomness” of mutation and recombination (both of which are based on random processes) and determines the direction in which the gene pool of a population changes. In the past, however, the process of selection has often been misinterpreted.

In part, this was because the British sociologist Herbert Spencer introduced the term “survival of the fittest” in his book “*Principles of Biology*” in 1864 [31]. Spencer drew parallels between his socio-economic theories and Darwin’s evolution theory, “This survival of the fittest, which I have here sought to express in mechanical terms, is that which Mr. Darwin has called ’natural selection’, or the preservation of favored races in the struggle for life” ([31], p. 444). Beginning with the fifth edition of “*The Origin of the Species*” [25], Darwin expanded the title of Chapter 4 to “Natural Selection, or the Survival of the Fittest”, because he was subjected to the accusation that the term “natural selection” personifies nature. Although Darwin’s idea of fitness refers to better adapted individuals for the immediate and local environment, currently, “fitness” is often misinterpreted as “being in the best physical shape”. Darwin’s idea that, in the struggle for existence, the better adapted individual survives, whereas its less well adapted competitors die out is not correct. Selection is usually expressed not in the extinction of competing con-specifics, but in differential reproductive and survival success [20,32]. These days, evolutionary biologists use exclusively “natural selection” and avoid “survival of the fittest”, because the latter suggests a continuity in evolution towards ever greater fitness. This would make today’s species “fitter” than extinct ones, which is not the case. Moreover, the term ignores the principle of sexual selection, where traits preferred by sexual partners are passed on.

### 2.4. Isolation

In biology, isolation refers to reproductive isolation, i.e., the interruption of gene flow between populations of the same species. Isolation occurs either by the spatial separation of populations of the same species (geographic isolation) or by the occupation of different ecological niches by populations of the same species within the same area (ecological isolation). Over time, these isolated populations are no longer able to produce fertile offspring with members of other populations. Isolation makes divergent evolution and the formation of genetic isolates, new subspecies, and ultimately, new species possible, as each population forms a closed genetic system with its own gene pool [33].

## 3. Evolution Strategies

### 3.1. Evolutionary Algorithms

The idea of the ES is that proposed solutions to an optimization problem are changed by random processes (analogous to biological mutation) and combined with each other (analogous to biological recombination) until the optimal solution is found by selection. To take the biological model into account, the elements of the search space in the ES are not called solution vectors, as is usual in mathematics, but individuals. Just as the individuals in living nature are better or less well adapted to their environment, some individuals in technology, i.e., technical objects that fulfill an optimization criterion better than others, are, for example, more stable, less expensive, or less material-intensive than the competing objects [20].

Although mathematical optimization methods fail as soon as the quality of an individual cannot be calculated with the help of a corresponding objective function, ESs can also be applied to problems in which quality can only be determined experimentally or subjectively. In any case, by analogy to biological selection, the less efficient solution proposals are discarded, and only the best solutions are retained [9,20].

Based on the biological model, the ES involves parents and offspring. The high level of abstraction of such an ES can be recognized from the fact that, unlike the biological model in which offspring usually have two parents, technical offspring can have one parent or several parents. The ES is a highly robust optimization method [22]. Despite the best offspring sometimes not being selected, the ES ultimately leads to an optimal solution, even in cases of incorrect evaluations (cf. Section 5.1) [20]. Several examples of the application of the ES in real-life engineering are given in Section 4.

### 3.2. Sequences of Operation in ES Symbolized by Playing Cards

**Figure 2:**
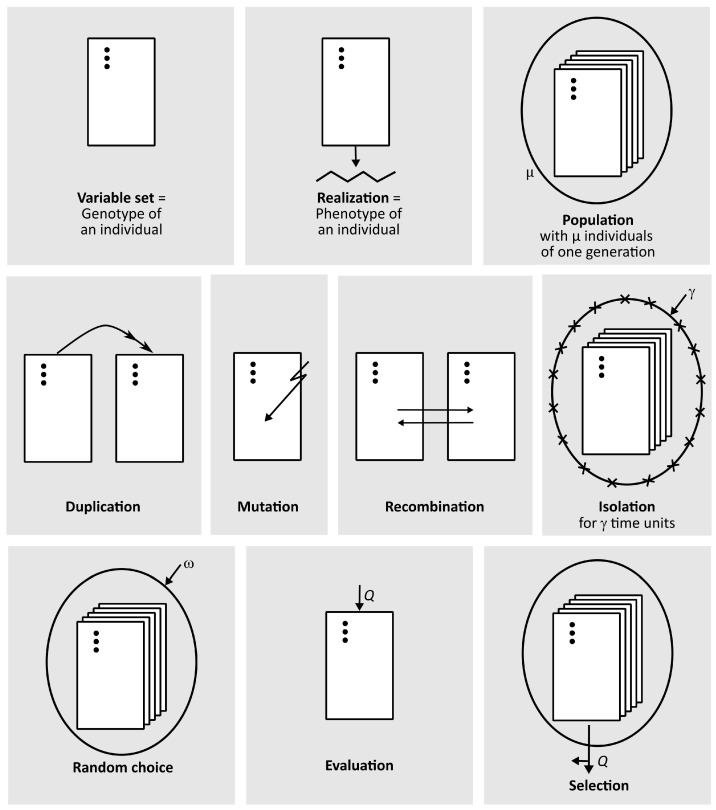
Graphical representation of basic elements for describing evolution strategies including symbols for individuals (i.e., variable set, realization, population), genetic operators (i.e., duplication, mutation, recombination, isolation), and selection and evaluation (i.e., selection, random choice, evaluation). The black dots on the cards indicate the variables, and the oval denotes that the cards are in a ballot box (adapted from [35,36]).

Because of the systematic and stepwise application of the principles of Darwinian evolution, ESs fall into the category of EAs [6,34]. On the other hand, ESs are a special case, because they are not a biomimetic product, but a universally applicable biomimetic method. Crucial to the success of a biomimetic innovation is the abstraction step, in which a common language is developed that can be understood by scientists from various disciplines. This common language of the ES includes the universal nomenclature for the variants of the ES (cf. Section 3.4, Equations (5)–(7)) and the symbolic playing cards. Figure 2 shows ten cards that are symbols for individuals, genetic operators, evaluation, and selection [35,36]. Various ESs are illustrated with playing cards in [35].

The ten symbols presented in Figure 2 denote the following in detail [14,35]:

1. Variable set: To reflect the biological model, the elements of the search space in the ES are not called solution vectors, as usual, but individuals. The object parameters are noted on the data card. The black dots represent a variable column. The variable set symbolizes the genotype of an individual in biology.
2. Realization: The zigzag line below a card indicates the realization of the object parameter values noted on it. In biology, the realization of an individual’s genetic information (genotype) forms its appearance or phenotype. In technology, the realization of object parameter values creates either a real technical object or a corresponding computer simulation.
3. Population: The variability of a population is symbolized by the different object parameter values of each individual. This is similar to the genotypes in a biological population. A set of cards surrounded by an oval indicates that the cards are in a ballot box.
4. Duplication: The double arrow indicates that the information on a data card is to be duplicated and transferred to a second card, resulting in a reproduction of the original individual. If the double arrow refers to a population, the entire record of the population will be copied.
5. Mutation: The zigzag arrow on a card indicates that the object parameter values noted on the card will be changed randomly. This change can affect all parameter values or it might be limited to a few pre-determined parameter values by a random process.
6. Recombination: Two opposing arrows between individual cards indicate that the numerical values of the individuals are randomly exchanged. Similar to the biological model, the ES distinguishes between dominant and intermediate recombination. In the case of dominant recombination, the value of each individual parameter of the offspring is statistically equally distributed to determine from which parent it is inherited. In the case of intermediate recombination, the offspring simply inherits the geometric mean of the parental values. Contrary to the usual biological model, the ES may not only involve two individuals, but might include several individuals in a recombination.
7. Isolation: The enclosure of a population by symbolic barbed wire indicates that the individuals in that population are isolated from the individuals outside the wire and, therefore, information cannot be exchanged between them. However, isolation only makes sense if it is lifted after a certain period of time (cf. Section 2.4). The letter γ is a measure of the duration of isolation. Whether the population is isolated from other populations for γ generations, γ seconds of processor time, or γ calls of the quality function must be determined in advance [9,16].
8. Random choice: A set of cards surrounded by an oval indicates that the cards lie within a ballot box. The arrow with an ω symbolizes that, depending on the context of the task, one or more cards will be randomly drawn from this ballot box to participate in the generation of the next generation’s offspring. Unless otherwise agreed, the random selection is based on the principle of equal statistical distribution. This means that each parent is equally likely to reproduce, and unlike the biological model, the selection of parents does not depend on the quality of the individuals. In other words, no sexual selection occurs in the ES. When several sets of cards lie within the ballot box, entire populations, rather than individuals, are selected according to this random principle.
9. Evaluation: The arrow with the letter *Q* indicates that the quality of an individual or of a population is recorded. The determination of the quality by means of experimental results, objective calculation, and subjective criteria (cf. Section 3.7) has the advantage that subsequent selection can be performed formally at the information level. The quality *Q* is inspired by the idea of fitness in biology.
10. Selection: The selection process is symbolized by a branching arrow with the letter *Q* next to it, indicating that the individuals in the ballot box are selected according to their quality *Q* (cf. Section 3.7). The selected individuals always have better quality values than those that are discarded. If several sets of cards lie within the ballot box, populations are selected according to this principle, not individuals [9,37].

### 3.3. Basic Version of Evolution Strategy

The basic version of the ES is carried out in the following iteration steps [7]:

0. Initialization: The individuals of the initial population are distributed as evenly as possible in the search space. Therefore, the initial population should be generated stochastically. Each individual represents a possible solution for optimizing the objective function (cf. Equations (1)–(4), and the fitness is calculated for each individual. The population size with μ individuals remains constant throughout the optimization process.
1. Recombination: Parents are selected stochastically with a “put-back” option that enables multiple drawing, and then recombined. Intermediate and dominate recombinations are possible (cf. Section 3.4).
2. Mutation: The offspring is mutated, evaluated, and stored in an intermediate population. Mutation is achieved by adding a mutation step size δ (cf. Section 3.5 and Section 3.6).
3. Selection: The iteration steps are repeated λ times, and then, the μ best individuals are selected for the next generation. We distinguish between the plus selection and the comma selection (cf. Section 3.4).
4. Termination: Iteration steps 1 to 3 are repeated until a termination criterion is met, such as the attainment of a specified maximum number of iterations or no further specified improvement of the solutions after several generations.

### 3.4. Variants of Evolution Strategy

When optimizing by the ES, several variants of the ES can be chosen that abstract biological evolution to various degrees. At the population level, a distinction can be made between the plus selection (cf. Equation (5), pronounced mu plus lambda membered evolution strategy), and the comma selection (cf. Equation (6), pronounced mu comma lambda membered evolution strategy). With plus selection, the parents are added to the ballot box and are, therefore, included in the selection. In contrast, with comma selection, the parents have a limited lifespan, are not added to the ballot box, and are, therefore, not included in the selection (cf. Section 3.7).

(5) (μ+λ)−ES

(6) (μ,λ)−ES

Whereas the letter μ indicates the number of parent individuals and the letter λ indicates the number of offspring in a generation, the plus or comma sign describes the way in which selection takes place. In both cases, offspring are produced by randomly selecting an individual from the μ parent individuals λ times in succession and minimally changing individual object parameter values by, for example, adding a random number (cf. Section 2.1). Following the evaluation of the individual offspring, in the case of plus selection, the offspring plus the parents are placed in a ballot box, and the best μ individuals are selected from the μ+λ individuals and declared the parents of the next generation. Therefore, a parent individual might be selected multiple times from the parent pool, and in the extreme case, all λ offspring descend from this single individual. In contrast, comma selection indicates that only the λ offspring compete with each other and that the best individuals become the parents of the next generation. In the latter case, as in biology, no potentially immortal individuals are present in the (μ,λ)–ES [20].

The ES variant shown in Equation (7) is a generalization of the plus selection and the comma selection and adds the mechanism of sexual recombination (cf. Section 2.2). The notation μ/ρ indicates that, in this ES variant, not one, but ρ parent individuals are involved in producing an offspring, and that each of these ρ individuals figuratively passes 1/ρ of its genetic information to the common offspring.

(7) (μ/ρ+,λ)−ES

In contrast to the typical biological model, the ES allows more than two individuals to be involved in a recombination. Thus, in the ES, λ times ρ individuals are selected simultaneously and with equal probability from the μ parent individuals and then recombined. In dominant recombination, a statistically uniform distribution determines from which parent individual each individual parameter value of the offspring is inherited. In intermediate recombination, the offspring simply inherits the arithmetic mean of the parental values.

### 3.5. Evolution Window

**Figure 3:**
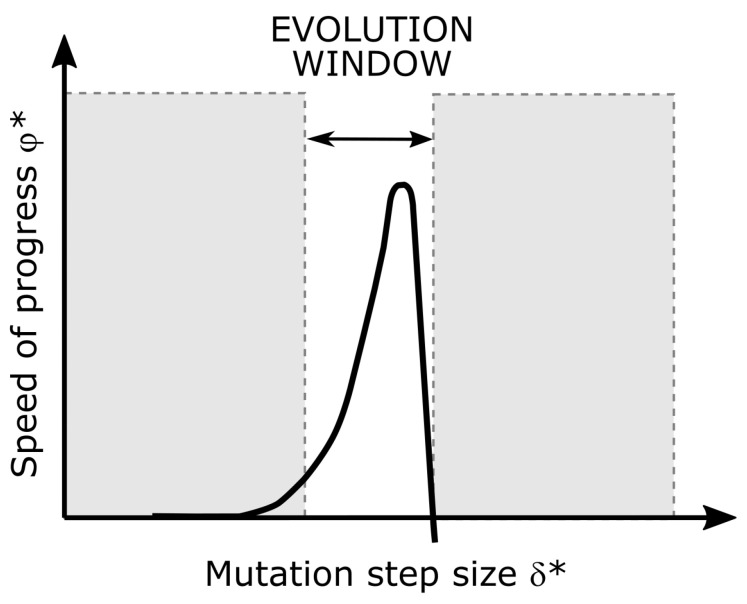
The evolution window results when the specific speed of progress φ* is plotted as a function of the specific step size δ*. The sharp maximum for the specific speed of progress is clearly visible. The calculation of φ* and δ* is given by Equations (11) and (12), respectively.

Ingo Rechenberg [35] introduced the concept and illustration of the evolution window into the ES. Figure 3 shows the speed of progress φ as a function of the mutation step size δ. In the ES, the mutation step size δ is a measure of the extent to which the mutation process changes the parental object parameter values $\chi_{P}$. Only if δ is chosen optimally is there a realistic chance of finding the optimum in a reasonable time. If the mutation step size δ is too small, stagnation occurs because the speed of progress φ is close to zero. If the mutation step size δ is too large, regression might occur because the speed of progress becomes negative [20].

#### 3.5.1. Speed of Progress

**Figure 4:**
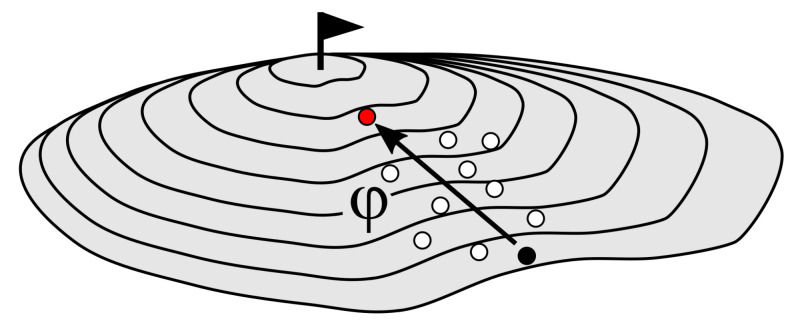
Mountain with several contour lines showing the speed of progress φ. The decisive factor is not the height gained on the mountain, but the distance traveled in the direction of the steepest slope. The climb starts with a parent that lies at a random position (black dot) and that produces ten offspring (nine white dots and one red dot). The offspring with the highest efficiency (red dot) will become the parent of the next generation.

The speed of progress φ (i.e., climbing speed) is a measure of the speed by which the ES moves towards the optimum in the quality landscape (Figure 4). The speed of progress φ is calculated as follows:

(8) $$\varphi =\frac{distance\;in\;direction\;of\;steepest\;slope}{number\;of\;generations}$$

As can be seen in Figure 3, it is advantageous to target the evolution window with the mutation step size at each point in the optimization. Thus, the step size must be constantly adapted during an optimization run (step size control), because the optimal step size has been shown to correlate with the local topology of the quality landscape. In summary, the more curved the contour line is at a point in the quality landscape, the smaller must the chosen mutation step size be for the evolution window to be continually targeted. In other words, the step size should become smaller and smaller as the distance to the optimum decreases, to avoid missing or skipping the optimum and ending up on the way back down [14].

#### 3.5.2. Mutation Step Size

Another central element of the ES is the so-called mutation step size δ, which is a measure of the extent to which the parental parameter values $\chi_{P}$ are changed by the mutation process. The mutation step size can be either global or individual. In both cases, small values of the mutation step size change the parental parameter values only slightly, whereas large values of δ result in correspondingly large changes. Since the global mutation step size is created by multiplying the mutation step size by the random vector *z*, all object parameters of the offspring $\chi_{O}$ are changed by the same amount (Equation (9)). In the case of individual mutation step sizes, the random vectors $z_{i}$ might differ, resulting in object parameters of the offspring $\chi_{O_{i}}$ that are changed by the respective amount (Equation (10)) [9,14,16].

(9) $$\chi_{O}=\chi_{P}+\delta \cdot z$$

(10) $$\chi_{O_{i}}=\chi_{P_{i}}+\delta_{i}\cdot z_{i}$$

Both the speed of progress φ and the mutation step size δ depend strongly on the topology of the quality landscape. As shown in Figure 4, a mountain in the quality landscape can be divided into an infinite number of contour lines, each of which has a different degree of curvature at each of its points. A measure of the degree of curvature is the mean radius of curvature Ω at that point. The following applies: the smaller that Ω is, the more that the contour line is curved at that point [9,38]. A prerequisite for keeping the evolution window is the calculation of the specific progress speed $\varphi^{*}$ (Equation (11)) and the specific mutation step size $\delta^{*}$ (Equation (12)) by using the radius of curvature Ω, because effective progress takes place only in the evolution window (Figure 3). These specific variables make it possible to adapt to the topology of the quality landscape and to avoid choosing steps that are too large in relation to the topology and, thus, skipping the peak and the local or even global optimum [9,14,37]:

(11) $$\varphi^{*}=\frac{\varphi }{\Omega }$$

(12) $$\delta^{*}=\frac{\delta }{\Omega }$$

The constant targeting of the evolution window means that an appropriate specific mutation step size must be found and kept approximately constant during the entire optimization run $\delta^{*}=\delta /\Omega \approx constant$. This, in turn, implies that the mutation step size δ must be based on the Ω measure: the more curved the quality landscape is at a point (i.e., the smaller the Ω value of the associated contour line), the smaller must the chosen mutation step size δ be so that the condition “$\delta^{*}=\delta /\Omega \approx constant$” continues to be satisfied. ESs that do not take this into account and work with a constant mutation step size during the entire optimization run will only lead to the goal if a step size is chosen by chance that always hits the evolution window without adapting to the Ω measure. Finding a mutation step size that targets the evolution window throughout the entire optimization run is virtually impossible for more complicated problems, since no recipe exists to guarantee that the evolution window will be met. The only chance of reaching the optimum as quickly as possible is to constantly adapt the step size to the topology of the quality landscape during the optimization run. In the ES, several adaptation techniques have been developed for this purpose, such as the “1/5 success rule” (cf. Section 3.6.1), “mutative step size control” (cf. Section 3.6.2), or “Covariance Matrix Adaptation” (cf. Section 3.6.3) [9,14,16,37,39,40,41,42].

### 3.6. Step Size Control

#### 3.6.1. The 1/5 Success Rule

The first concept of step size control, the so-called “1/5 success rule”, was derived theoretically by Ingo Rechenberg using a (1+1)–ES as an example. He was able to show that the mutation step size is optimally chosen if, on average, every fifth mutation is successful [9,39]. This is described by the success probability $W_{e}$ (Equation (13)). “Successful” in this context means that the offspring carrying the mutation has a better value of the quality function than its parent.

(13) $$W_{e}=\frac{successful\;mutation\;steps}{total\;of\;mutation\;steps}\approx \frac{1}{5}$$

If $W_{e}<1/5$ during an optimization run, the step size should be decreased to increase the success probability. On the other hand, the mutation step size should be increased if $W_{e}>1/5$. Since the optimal value $W_{e_{opt}}=1/5$ was determined purely theoretically, it is only justified, “if no disturbances distort the quality” ([9], p. 367). However, disturbances such as measurement errors must be taken into account, especially during experimental optimization. Experience has shown that, in this case, we should aim at a value of $W_{e}>1/10$. In addition to the fact that the 1/5 success rule is only a rough guideline and can fail, for example, if the quality function of an optimization problem has discontinuities in the first derivative, this type of step size control has the disadvantage that it must be controlled from the outside, i.e., by the experimenter [8,14].

#### 3.6.2. Mutation Step Size Control

Obviously, it would be advantageous if the mutation step size could adapt itself to the local topology of the quality landscape during an optimization run. A step size control that satisfies this requirement is the “mutative step size control” [8,9,37]. Like the object parameters, the step size mutates and is passed to the next generation. A (1,9)–ES with mutative step size control is used in the educational module “Optimization of a Milk Carton” (cf. Section 5.1).

We start with one parent individual and an arbitrary mutation step size δ, which should be chosen from experience so that the largest possible mutation crosses at most one third of the parameter space. The λ offspring are always generated according to the same scheme: first, the parental mutation step size $\delta_{P}$ must be varied in order to be able to mutate the object parameter χ with the resulting offspring step size $\delta_{O}$. The newly added variable ξ is a measure of the extent to which the parental mutation step size is varied. Thus, Equation (14) applies to the offspring λ:

(14) $$\delta_{O_{\lambda }}=\xi_{\lambda }\cdot \delta_{P}\Rightarrow \chi_{O_{\lambda }}=\chi_{P}+\delta_{O_{\lambda }}\cdot z_{\lambda }$$

The step size change factor α is used to both increase (Equation (15)) and decrease (Equation (16)) the parental mutation step size $\delta_{P}$, with an empirical value of 1.3 for alpha being determined experimentally [9].

(15) ξ=α=1.3

(16) ξ=1/α=1/1.3≈0.77

The mutation process is followed by the evaluation. The quality *Q* of an offspring is determined solely by the object parameters and not by the mutation step size. The λ offspring are selected solely on the basis of their quality. Since the mutation step size belongs to the respective individual, it is also indirectly selected [14]. In general, the best quality values are provided by those offspring whose step size is best adapted to the topology of the quality landscape, and the course of the optimization strongly depends on the mutation step size chosen at the beginning [9]. For the sake of completeness, it should be mentioned here that the mutation step size of an offspring in the case of recombination is calculated as the geometric mean of the ρ step sizes of the parents [16].

Obviously, mutative step size control only works for global step sizes. However, an efficient step size control should adapt both individual and global step sizes. Currently, the only step size control that satisfies this requirement is the Covariance Matrix Adaptation (CMA).

#### 3.6.3. Covariance Matrix Adaptation

The Covariance Matrix Adaptation was introduced in the mid-1990s by Gawelczyk, Hansen, and Ostermeier [41] and remains the state of the art in optimization by the evolution strategy (CMA-ES). This type of step size control is also based on the idea of increasing the probability of previously successful mutation steps. However, this does not mean that the mutation step δ is mutated and selected directly, as in the mutation step control, or indirectly, as in the 1/5 success rule. Instead, in the CMA-ES, the probability distribution underlying the optimization, which is used to generate the random vector *z* for changing the parental object parameter values, is adapted as a whole to the local topology of the quality landscape. Adaptation to the local topology is achieved by changing the associated covariance matrix based on an evaluation of the selected mutation steps. In this context, the mutation distribution preferentially generates random vectors that are similar to the vectors selected in the last generation to change the parent object parameter set. For this purpose, only the previous covariance matrix has to be added to the covariance matrix of the selected random step [14,16,40]. In probability theory, covariance is a measure of the relationship of two random variables, and a covariance matrix is a matrix that contains all pairwise covariances of the components of a random vector. We have used the CMA-ES for step size control in the EvoBrach software (Section 5.2).

**Figure 5:**
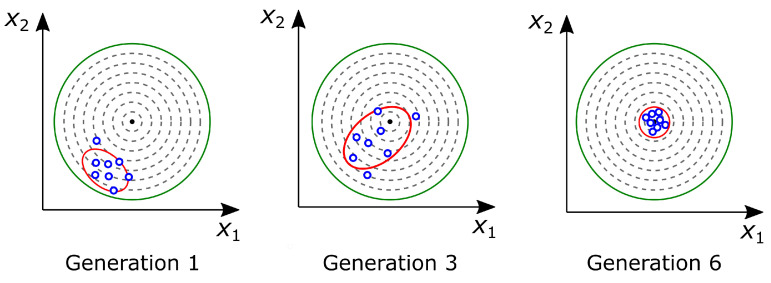
Two-dimensional plot showing the actual optimization run with the Covariance Matrix Adaptation evolution strategy (CMA-ES) on a simple two-parameter problem. In a fitness landscape (green lines and gray dashed lines), a generation of points (blue dots) is produced with the mean in the center of the bounds determined by the standard deviation (red lines). Within a few generations, the population becomes concentrated around the global minimum in the center of the plot (black dot). Adapted from [42].

Corpus et al. [42] employ the CMA-ES for the two-parameter minimization problem schematically depicted in Figure 5. In the first generation, the CMA-ES produces a generation of points (blue dots) whose parameter values are assigned by an initial Gaussian distribution. The mean is centered on the given elliptical boundaries, whose radii are determined by the standard deviations of the distribution (red lines). The CMA-ES ranks the points according to their nearness to minimizing the objective function (black dot in the center of the fitness landscape). The CMA-ES then shifts the distribution towards the points with the best ranking in terms of objective minimization. As the points come closer and closer to the global minimum, the CMA-ES starts to reduce the standard deviation and converges to the global minimum. The process is finally stopped when the standard deviation of the points reaches a predetermined small value, indicating that all generated points are converging to the global minimum [42].

### 3.7. Selection Criteria

Individuals or populations in the ES are evaluated in three ways: (1) the quality *Q* is determined experimentally, or (2) *Q* is calculated using a fitness or quality function, or (3) *Q* is determined according to subjective criteria. The arrow with the letter *Q* on the card indicates that, in these three cases, the quality of an individual is recorded on the corresponding card. This process has the advantage that selection can be performed formally at the information level [14].

In the simplest case, the quality *Q* of a population could be the geometric mean of the individual qualities. However, it is much more effective to evaluate populations according to criteria other than the individual members. This is the only way to take into account the biological phenomenon in which, during evolution, traits can arise that are neutral or even detrimental to individuals, but beneficial to the population as a whole [9,16].

**Figure 6:**
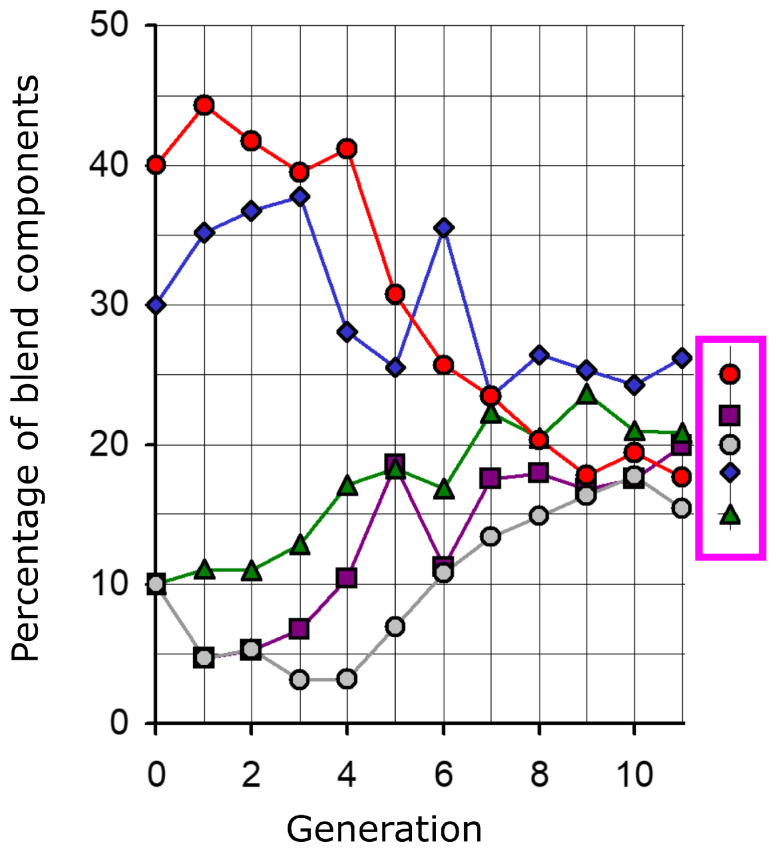
Result of the optimization of the taste of a coffee that is a blend of five coffee types. Based on five coffee blend components, a (1,5)–ES with subjective selection was used for the optimization. After eleven generations, the taste of the coffee did not differ considerably from that of the target coffee. The usual composition of the target brand coffee is indicated by the symbols to the right of the diagram in the magenta box and differs considerably from the composition found using the ES (Adapted with permission from [16]).

A special feature of the ES is subjective optimization, which allows one subjectively to decide which offspring are of the best quality and, thus, form the starting point for further evolutionary loops. A prime example is the optimization of the taste of coffee, which is a blend of five different types of coffee. Because coffee is a natural product, the composition of the coffee blends must be redefined each year to ensure that the target coffee has the characteristic taste that consumers expect. Based on a parent coffee blend whose five components were deliberately defined by the experimenter far from the target coffee blend, a (1,5)–ES was applied for the optimization (Figure 6). The parental coffee blend differed considerably from the blend normally used for the target coffee. Each of the five offspring coffees was created by randomly modifying the parental blend components with an algorithm described in Section 5.1. Once the five descendant coffees had been obtained, three professional coffee testers determined the best offspring coffee by tasting, namely the one that the testers found to be the closest to the target coffee taste. After just eleven generations, no discernible difference could be found in taste between the selected offspring coffee and the target coffee [43,44].

## 4. Application of Evolution Strategy in Technology

**Figure 7:**
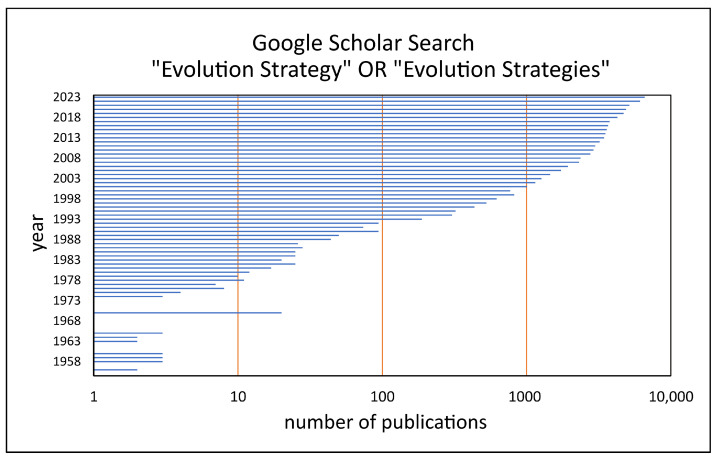
Number of publications in the Google Scholar database with a total of 79,529 articles. A significant increase of articles including the words “evolution strategy” OR “evolution strategies” is found for the period from 1955 to 2023. Please note that the x-axis has a logarithmic scale. Raw data retrieved from Google Scholar on 8 May 2024, cf. Supplementary Materials File S5.

Over the last 60 years, early evolutionary methods and their modifications have been further developed in a large number of research projects, and scientific results have been published in peer-reviewed journals (Figure 7) and have been used in real-life engineering applications [2]. Examples are the optimization of an artificial plate flexible in five positions with the aim to find the shape exhibiting the minimum drag [35], the cost efficiency analyses of steel frameworks for the economical design of multi-story buildings [45], the realization of dynamically adaptive batteries exploiting an on-line steady-state ES [46], the enhancement of the performance of a multi-objective evolutionary algorithm that provides sanitary sewer overflow reduction and that improves the convergence rate by approximately 70% over the tested alternative algorithms [47], and the application of a (1,5)–ES with subjective selection to determine the ratio of different coffee types needed to obtain the taste of a special target mixture [43,44].

Like its biological model, the ES uses random processes to achieve the optimum. Although Ingo Rechenberg has argued that, “the Evolution Strategy […] is the universally best of all optimization strategies” (translated from German by the authors: “die Evolutionsstrategie […] ist die universell beste aller Optimierungsstrategien”; [9] p. 24), Nissen counters that, “evolutionary algorithms should not be misunderstood as a `panacea’ ” (translated from German by the authors: “Andererseits sollten Evolutionäre Algorithmen aber nicht als “Allheilmittel” missverstanden werden”; [13] p. V of the Preface).

If an optimization problem can be formulated mathematically and its structure allows the use of classical mathematical optimization methods, then the computationally intensive ES should not be used. On the other hand, this method is extremely good at solving problems for which classical optimization strategies fail. However, the ES is particularly suitable as an optimization method in situations in which the quality of an individual can only be judged experimentally or subjectively. According to Slowik and Kwasnicka [2], the three most popular areas of application for the ES are computer science artificial intelligence, engineering electrical electronics, and computer science theory methods.

## 5. Educational Modules on Evolution Strategy

Today’s students are growing up in a world that is more scientific and technical than ever before. Thus, modern education should take this into account and aim to teach scientific and technical ways of thinking and working, in addition to purely providing biological and technical knowledge. Biomimetics is particularly suited to this because, “biomimetic thinking and action develops the ability of students to think in complex and networked ways” [14,48,49].

In Supplementary Materials Files S1–S3, we present three educational modules that can be directly used by lecturers, teachers, and students from high schools, colleges, and universities in order to achieve a better understanding of the ES. Each module is divided into five work sheets. Work sheet (1) is a general “Information on evolution strategy” that is identical for all modules. The content overlap with the article is intentional, as we do not expect students to read the entire article, but rather to study work sheet (1) as an introduction to the topic of ESs and as a basis for the experiments. The other work sheets of the educational modules are thematically different and provide details on (2) specific information about the optimization problem, (3) the experiments to be performed, (4) the evaluation of the data, and (5) solutions to all tasks and a discussion of the results.

### 5.1. Educational Module: Optimization of a Milk Carton

The educational module “Optimization of a Milk Carton” is provided in Supplementary Materials File S1 including the work sheets (1) Evolution Strategy: General information, (2) Information: Optimized material consumption of a milk carton, (3) Experiment: Experimental setup and performance of the experiment, (4) Evaluation: Analysis of the data, and (5) Solutions: Answers or individual solutions to all tasks and a discussion of the experimental results [20].

**Figure 8:**
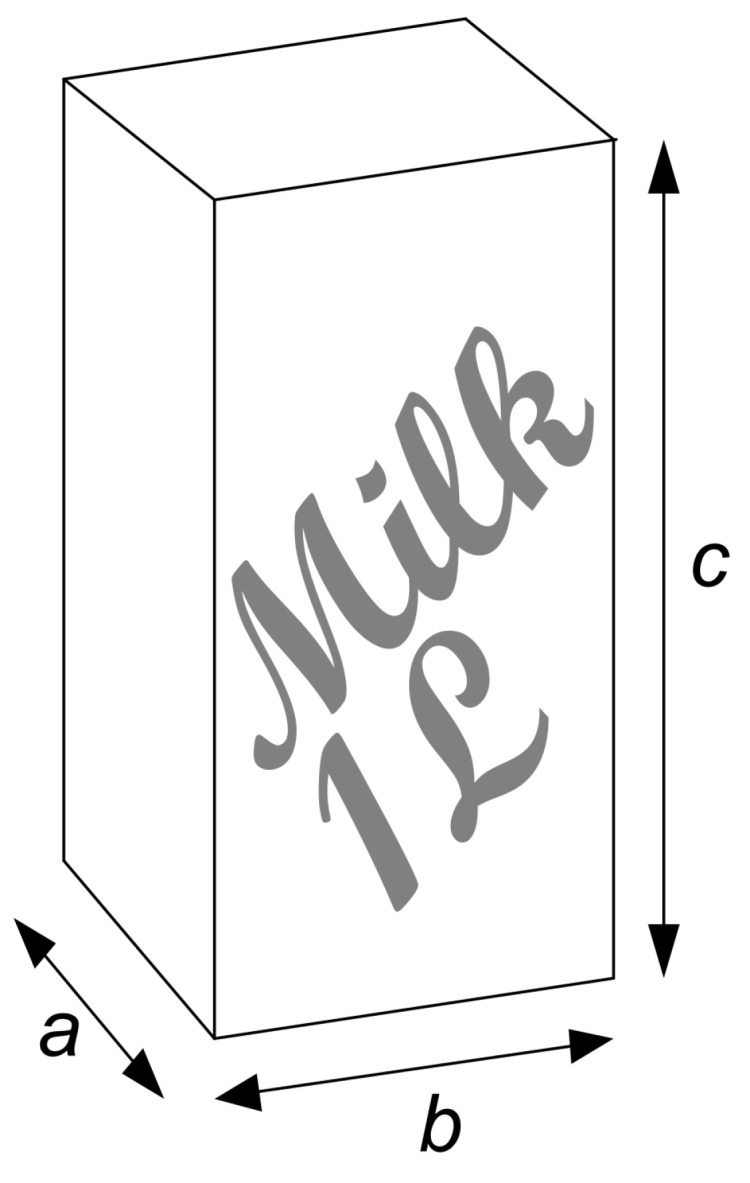
Cuboid milk carton. At the beginning of the (1,9)–ES, the side lengths of the milk carton are *a* = 5 cm, *b* = 10 cm, and *c* = 20 cm, and thus, it has a quality of Q=2(ab+ac+bc) = 700 cm^2^ (generation 0).

There are countless ways to package a liter of milk into a cuboid. However, to save material costs and avoid unnecessary waste, milk cartons should always be produced with a minimum amount of material. Whether commercially available milk cartons are optimal in this respect can be easily tested with the optimization experiment presented here. The preliminary considerations can be worked out in a discussion with the students. The following questions should be addressed (see work sheet “Information”):

- What is the goal of the optimization?
  The primary optimization goal is to minimize the material consumption of a milk carton while maintaining the same volume. If, as in this case, glued surfaces and folds are not taken into account, this means that the surface area of the milk carton pack should be minimized (cf. Equation (1)).
- What changes can be made to the optimization object to achieve the optimum?
  The surface area of a cuboid can be influenced by varying its side lengths (Figure 8).
- How can a variable object be efficiently evaluated?
  The quality *Q* of a cuboid can be calculated as Q=2(ab+ac+bc), where *Q* is the surface area and *a*, *b*, and *c* are the side lengths of the milk carton.
- Which constraints limit the optimization?
  The milk carton should still hold a volume of 1 L (a·b·c = 1000 cm^3^ = 1000 mL). In addition, the side lengths must never become negative or zero (a,b,c>0 cm).
- How many input variables does this optimization problem have?
  This is an optimization problem with two input variables. There will always be offspring violating the constraint a·b·c = 1000 mL if all three side lengths are varied.

**Figure 9:**
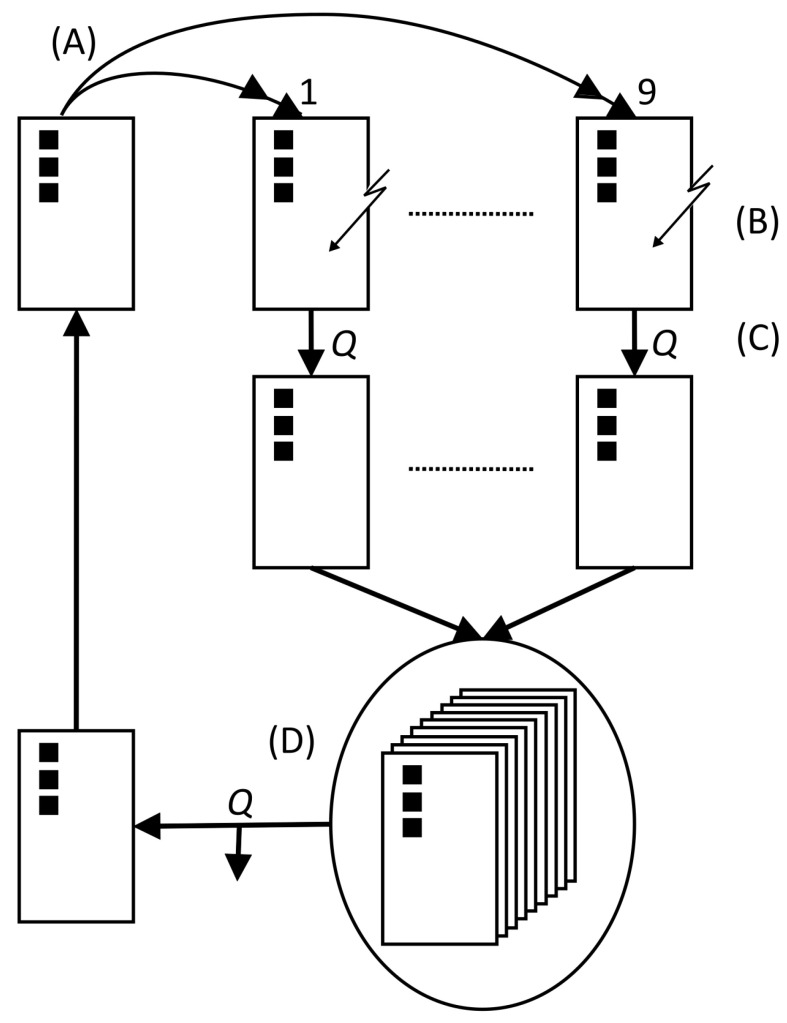
Graphical representation of the (1,9)–ES used for the optimization of a milk carton with minimal material consumption. An individual is symbolized by the variable set card, on which the object parameter values are written, indicated by the black squares. (A) duplication, (B) mutation, (C) evaluation = determination of the quality *Q*, and (D) selection = choosing the individual with best quality.

The milk carton with the minimum material consumption should be determined using a (1,9)–ES (Figure 9) with mutative step size control. We have designed the module so that a class of 18 students can ideally be divided into nine teams of equal size, with 2 students in each team working together. In this case, each team generates one of the nine offspring. If necessary, the number of teams and team members can be adapted to the number of students in the class. Each team needs one dice, one pocket calculator, and several copies of the work sheet “Template” provided in work sheet “Experiment”.

**Figure 10:**
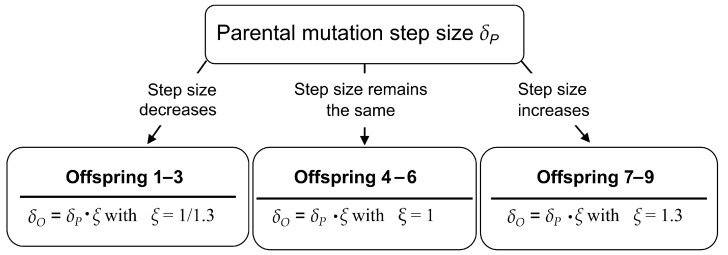
Mutative step size control of the (1,9)–ES to optimize the milk carton. Since the mutation step size of the offspring is calculated as $\delta_{O}=\delta_{P}\cdot \xi$, the mutation step size $\delta_{O}$ decreases if ξ<1.0, increases if ξ>1.0, and remains unchanged if ξ=1.0.

The optimization is started with a milk carton whose dimensions correspond approximately to those of a standard milk carton with a rectangular base (Figure 8). Since an ES with mutative step size control is used, not only a starting individual, but also a starting mutation step size must be specified. In this case, it proved useful to start with the empirically determined step size δ = 0.6. However, the ES can also be performed with a starting step size δ = 0.5 or δ = 0.4. Figure 10 shows the mutative step size control of the (1,9)–ES to optimize the milk carton. Although three of the nine offspring adopt the parental step size unchanged with ξ=1.0, the remaining six offspring are equally generated with a reduced (ξ<1.0) or increased (ξ>1.0) mutation step size. In this case, we have a distribution of 3-3-3 for nine teams and nine offspring. Even with different numbers of teams or team members, the division of the generated offspring should always be such that increases and decreases in step size are equally likely. For example, for 11 offspring, a distribution of 4-3-4 should be selected, for 7 offspring a distribution of 3-1-3, and for 4 offspring a distribution of 2-0-2.

**Figure 11:**
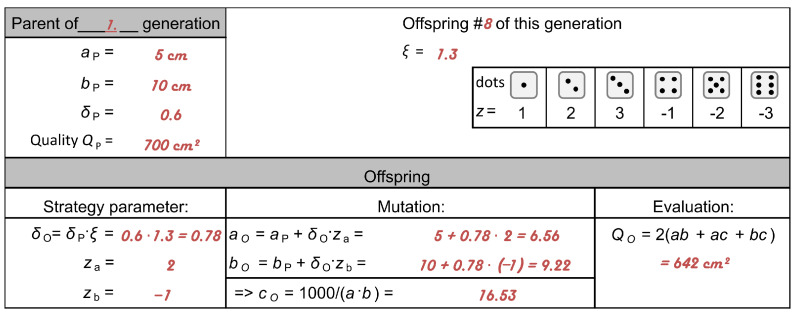
Initialization of the milk carton optimization inserted in the “Template” provided in the work sheet “Experiment”. In this example, two dots were obtained in the first throw with the dice, resulting in $z_{a}=2$, and four dots were obtained in the second throw with the dice, resulting in $z_{b}=-1$. Numbers in red refer to milk carton generation 0.

Each team produces the same offspring in each generation (e.g., offspring 8) and records the data using the “Template” provided in work sheet “Experiment”. As shown in Figure 11, the optimization starts with the values of the milk carton from generation 0 with $a_{P}$ = 5 cm, $b_{P}$ = 10 cm, $\delta_{P}$ = 0.6, and quality $Q_{P}$ = 700 cm^2^ (cf. Figure 8). According to Equation (10), the offspring parameters are mutated by multiplication with a factor *z*, which is a random value generated with a dice. Each team rolls the dice twice. The dot number of the first throw generates the random factor $z_{a}$ for the variation of the parental side length $a_{P}$, and the dot number of the second throw generates the random factor $z_{b}$ for the variation of the side length $b_{P}$. To ensure that the side lengths become longer and shorter with equal probability, the dot numbers 1, 2, and 3 correspond to the random factors *z* 1, 2, and 3, and the dot numbers 4, 5, and 6 correspond to the random factors *z*−1, −2, and −3. According to the equations in the “Template” provided in work sheet “Experiment”, first, the side lengths $a_{O}$ and $b_{O}$ are calculated for the offspring, and then, its side length $c_{O}$ and its quality $Q_{O}$ are calculated. From the nine offspring, the offspring with the smallest value for the surface area is selected as the parent of the next generation, and the calculation is continued using its values. The above steps are repeated until the quality of the selected offspring is “sufficiently good” or has not changed markedly in the last three to five generations.

**Figure 12:**
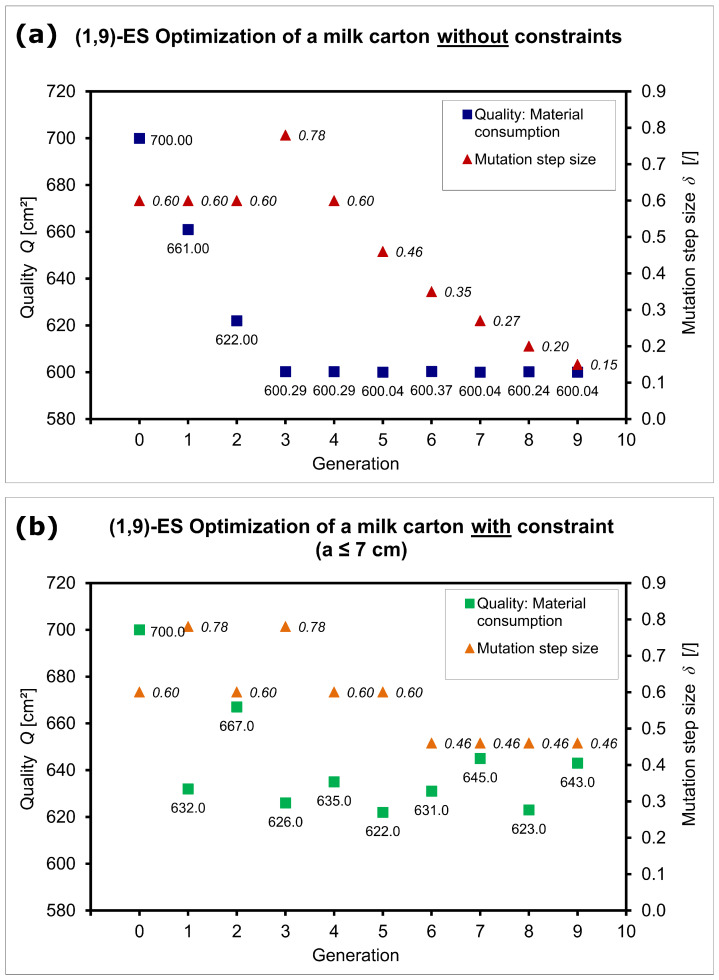
Results of the milk carton optimization with the (1,9)–ES displaying the quality *Q* in terms of material consumption [cm^2^] and the mutation step size δ [/]. Individual solutions are shown for the optimization (**a**) without constraints and (**b**) with the constraint a≤ 7 cm. The optimization without constraints creates a cube, whereas the optimization with the constraint creates a cuboid.

Without further constraints, a cube with a side length of 10 cm is obtained as the milk carton with the lowest material consumption with a quality of $Q_{cube}$ = 2(100 cm + 100 cm + 100 cm) = 600 cm^2^. Therefore, “sufficiently good” means a quality value between 600 cm^2^ and 600.20 cm^2^ (Figure 12a). If we introduce the constraint that a side must not be longer than 7 cm, because, for example, the milk carton should fit into the refrigerator door, then a cuboid is created (Figure 12b).

The optimization method ES is highly robust with respect to mistakes. Mylo and Speck [22] present comparative results of the milk carton optimization when the best offspring is always selected or when a mistake is made in generation 5 and only the second best offspring is accidentally selected (cf. Figure 7 in [22]). Despite this mistake, the optimization with the ES is robust and approaches the minimum material consumption.

### 5.2. Educational Modules: Fastest and Shortest Marble Track and Various Marble Track Shapes

The educational module “Fastest and Shortest Marble Track” is provided in Supplementary Materials File S2 including the work sheets (1) Evolution Strategy: General information, (2) Information: The brachistochrone problem, (3) Experiment: Construction of a marble track, experimental setup, and experiments, (4) Evaluation: Analysis of the data, and (5) Solutions: Answers or individual solutions to all tasks and a discussion of the experimental results [50].

The educational module “Various Marble Track Shapes” is provided in Supplementary Materials File S3. The module is based on the EvoBrach software, a demonstration program that has been developed for students and that simulates the use of the ES to compare the running times of a marble on a straight line, a parabola, and a brachistochrone curve. Supplementary Materials File S3 includes the work sheets (1) Evolution Strategy: General information, (2) Information: The brachistochrone problem, (3) Experiment: Introduction to the software “EvoBrach” and digital experiments, (4) Evaluation: Analysis of the data, and (5) Solutions: Answers or individual solutions to all tasks and discussion of the experimental results [50]. We provide the EvoBrach.exe file in Supplementary Materials File S4.

In June 1696, the Swiss mathematician Johann Bernoulli publicly presented in *Acta Eruditorum*, the first German scientific periodical, the so-called brachistochrone problem to, “the wisest mathematicians of the whole world” [51]. Originally written in Latin, his introduction to the problem is translated as follows [52], “I, Johann Bernoulli, address the most brilliant mathematicians in the world. Nothing is more attractive to intelligent people than an honest, challenging problem, whose possible solution will bestow fame and remain as a lasting monument. Following the example set by Mersenne, Pascal, Fermat, etc., I hope to gain the gratitude of the whole scientific community by placing before the finest mathematicians of our time a problem upon which as upon a touchstone could test their methods and the strength of their intellect. If someone communicates to me the solution of the proposed problem, I shall publicly declare him worthy of praise.” A further translation of the problem that he posed is as follows [52], “Given two points A and B in a vertical plane, what is the curve traced out by a point acted on only by gravity, which starts at A and reaches B in the shortest time?”. Five solutions to Johann Bernoulli’s problem were published, in January 1697 by Isaac Newton [53] and in the May 1697 issue of *Acta Eruditorum* by Gottfried Wilhelm Leibniz [54], by Johann Bernoulli himself [55], by his eldest brother Jakob Bernoulli [56], and by the Marquis de l’Hôpital [57].

Determining both the shortest path (=a straight line) and the fastest path (=the brachistochrone curve) from the starting point A to a lower end point B (with B not being directly under A) are optimization problems. The word “brachistochrone” derives from the Greek words *brachýs* meaning “short” and *chrónos* meaning “time”.

**Figure 13:**
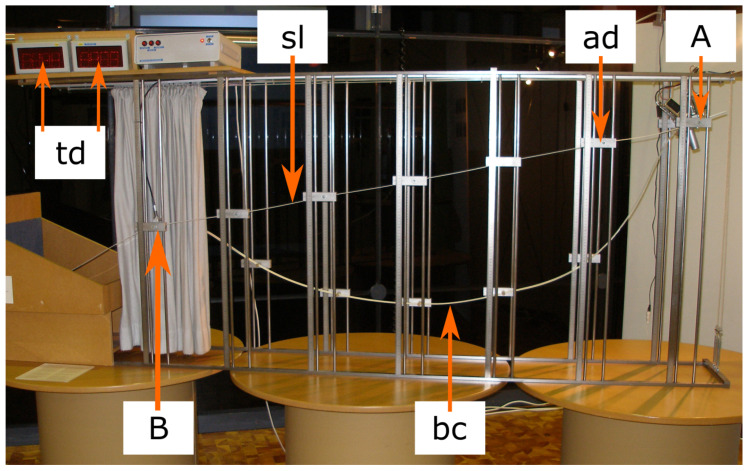
Marble track in the biomimetics exhibition of the TECHNOSEUM–Landesmuseum für Technik und Arbeit in Mannheim, Germany, 2007. Visitors can start one marble on the straight line (sl) and one on the brachistochrone curve (bc). The running times of the marbles from the starting point (A) to the end point (B) are recorded by light barriers and shown on the time displays (td). Different curve generations can be generated using the five height-adjustable supports (ad), and the shape with the best quality in terms of the shortest running time can be found using the evolution strategy.

Some science centers, such as the Mind Museum in Taguig City, Philippines, and the DYNAMIKUM in Pirmasens, Germany, have built several slides with different shapes side by side. One slide is always a straight line, and another is a brachistochrone curve. This allows visitors to experience the difference in time and acceleration for themselves. Other science centers offer a hands-on experiment in which one marble travels the shortest path and another marble travels the fastest path in parallel on two tracks. If the marble track is long enough, the difference in the time it takes for the marbles to reach the goal (point B) can be seen with the naked eye. In most marble tracks used for visitor experiments in science centers, the straight line and the brachistochrone curve are fixed, and the running time of the marbles can be compared. Other marble tracks, such as the those at the TECHNOSEUM in Mannheim, Germany (Figure 13), and at the Eugenides Foundation in Athens, Greece, are built so that the shape of the curve can be changed. This allows the visitors to generate several generations of curves and to find the optimal curve shape with the shortest running time by using the ES.

The following applies: the faster the marble reaches the end point B, the higher the quality of the curve. Of course, the height of the start and end of the curve are not changed during the entire optimization process. Starting with an arbitrary poor curve (=parent = generation 0), the brachistochrone curve can now be determined experimentally using the ES as follows:

1. The ES on which the optimization is based must be defined, e.g., (1,3)–ES with mutative step size control.
2. Changing the parental support heights by small random values calculates the support heights of the offspring curves.
3. All offspring curves are realized one after the other, and their running times are determined.
4. The offspring with the smallest running time becomes the parent of the next generation.
5. Steps 2 to 4 are repeated until a predefined termination criterion is met.

Although the experimental solution of the brachistochrone problem leads to the goal, it has a decisive disadvantage: it is too time-consuming and, therefore, unsuitable for use in high schools, colleges, and universities. Therefore, we present the construction plans of a marble track with a fixed straight line and a fixed brachistochrone curve in Supplementary Materials File S2. If the marble track is long enough, the difference between the running times of the shortest and the fastest track can be seen with the naked eye. With the construction of the marble track, students will have a permanent structure that can be used for demonstration purposes outside the ES context. Using the work sheets in the educational module, students will learn about the physical principles that explain why the shortest path is not the fastest path. The module requires students to be familiar with mathematical equations and to understand the theorem of rays.

**Figure 14:**
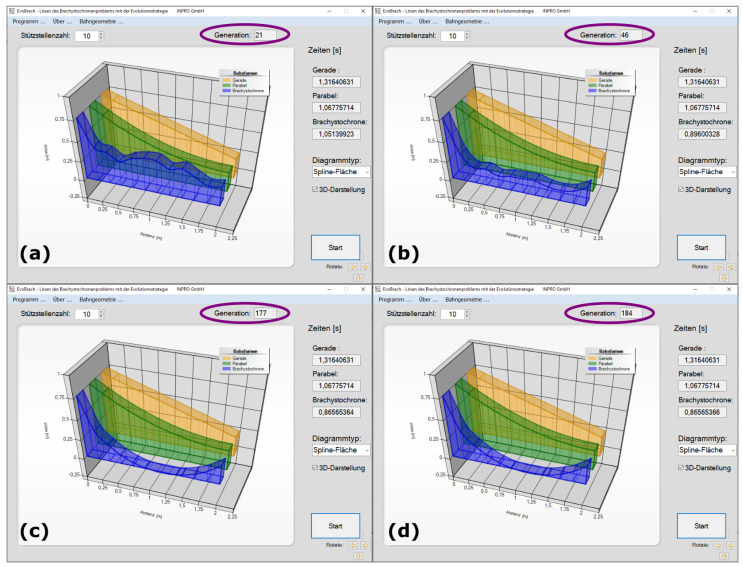
Screenshots of the German EvoBrach software comparing the running time in seconds with 8 digits after the decimal comma (=Zeiten [s]) between the yellow straight line (=Gerade), the green parabola (=Parabel), and the blue brachistochrone curve (=Brachystochrone). Since this is German software, the decimal points are commas. For all experiments, 10 support points (=Stützstellenzahl) were selected. (**a**,**b**) The experiments were stopped during the optimization processes. (**c**,**d**) The optimization processes were completed up to the termination criterion. The optimization of the brachistochrone curve using the evolution strategy is indicated in (**a**,**b**) by the random curve shapes that it takes during the optimization and in (**c**,**d**) by the different number of generations (=Generation) that it takes to complete the optimization processes.

To overcome the disadvantage of a time-consuming optimization of track shapes using the ES, we have also developed the freely available EvoBrach software using the ES for finding the brachistochrone curve within a few seconds (Figure 14). The EvoBrach.exe file together with the German EvoBrach manual is provided in the Supplementary Materials File S4. At the start of the optimization, the user can select the number of support points (=Stützstellenzahl) that divide the horizontal distance between the starting point and the end point into equidistant sections. Independent of the number of support points, a straight line (yellow) and a parabola (green) appear after pressing the start button. Both curves are fixed and serve as comparison paths during optimization. Only the blue curve is optimized using a (1,10)–ES with the adjustment of the covariance matrix. By randomly changing the height of each support point, 10 offspring are generated. The offspring with the lowest running time is the parent of the next generation. This process continues until the termination criterion is met: if there is no change in the eighth decimal place of the running time in seconds for more than ten generations, the optimization stops.

## 6. Conclusions

Finding an optimal solution is a challenge in both engineering and biological evolution. In this context, algorithms used for optimization in mathematics are systematically applied in engineering. However, mathematical optimization methods fail as soon as the quality of a solution cannot be calculated with the help of a corresponding objective function. This is where ESs come into play, because they can also be applied to problems in which quality can only be determined experimentally or subjectively. The ES is a biomimetic optimization method, abstracted from the functional principles of recombination, mutation, isolation, and selection by Darwinian evolution. Unlike engineers who design with a goal in mind, biological evolution has no predetermined target. Instead, biological structures have evolved through trial and error, resulting in the diversity of plants and animals that are adapted to their habitat on our planet. The article introduces both the functional principles of biological evolution and the mechanisms of the ES. Furthermore, the understanding of the minimization and maximization problems is deepened and the view of the difference between optimized and optimal solutions is sharpened.

Lecturers, teachers, and students from high schools, colleges, and universities can use the educational modules to improve their knowledge of the application of the ES to minimization problems. In the “Optimization of a Milk Carton” module, we search for the most material-efficient packaging for a liter of milk. Of particular pedagogically value, the side lengths of the milk cartons are “mutated” with the help of a dice. The dice illustrates the “trial and error” approach of biological evolution, and the graphical representation shows a significant reduction in surface area after only a few generations. The other two educational modules focus on the brachistochrone problem, i.e., finding the shortest path between two points. Experiments can be used to verify that the shortest path (=straight line) is not the fastest path (=brachistochrone). The self-made marble track from the module “Fastest and Shortest Marble Track” can be used for analog experiments. Digital experiments can be performed in the module “Various Marble Track Shapes”, based on the EvoBrach software, which uses the ES to develop the fastest path. Both modules deepen the understanding of the physical bases of potential and kinetic energy and of the ES. In conclusion, the introduction to the biomimetic method of the ES and the deepening of knowledge within the educational modules should lead to a better comprehension of both optimization problems and biological evolution.

## Figures and Tables

**Table 1:** Comparison of adaptation in biological evolution and the evolution strategy used in technology (from [22]).

	Biological Evolution (Biology)	Evolution Strategy (Technology)
Subject	Living being	Object to be optimized
Mutation	Random change of genetic information	Random change of input variables (i.e., object parameters)
Recombination	Reshuffling of parental genetic material (e.g., meiosis)	New combination of parental object parameters
Selection	Selection of those individuals with the best fit to the natural environment	Selection of those individuals that best meet the optimization criterion
Result	Adapted organism	Optimized object

## Data Availability

All relevant data are included within the article and its Supplementary Material Files S1–S5.

## Supplementary Materials

The following Supporting Information can be downloaded at: https://www.mdpi.com/article/10.3390/biomimetics9070439/s1, File S1: Educational Module “Optimization of a Milk Carton”; File S2: Educational Module “Fastest and Shortest Marble Track”; File S3: Educational Module “Various Marble Track Shapes”; File S4: EvoBrach.exe file and EvoBrach manual (in German); File S5: Raw data retrieved from Google Scholar.

## Abbreviations

The following abbreviations are used in this manuscript:

CAM	Covariance Matrix Adaptation	
EA	evolution algorithm	
EP	evolutionary programming	
ES	evolution strategy	
GA	genetic algorithms	
*g*	parameter *g*	
*h*	parameter *h*	
*O*	offspring	
*P*	parent	
*Q*	quality	
*z*	random factor	
α	(pronounced alpha)	change factor of step size
γ	(pronounced gamma)	time unit of isolation
δ	(pronounced delta)	mutation step size
λ	(pronounced lambda)	number of offspring in a generation
μ	(pronounced mu)	number of parent individuals selected
ξ	(pronounced xi)	variation of mutation step size
ρ	(pronounced rho)	number of parents involved in recombination
χ	(pronounced chi)	object parameter
Ω	(pronounced Omega)	radius of curvature
ω	(pronounced omega)	random drawing from ballot box
