# Supplementary material for: The Basics of Evolution Strategies: The Implementation of the Biomimetic Optimization Method in Educational Modules
Source: Biomimetics (Basel). 2024 Jul 18;9(7):439. doi: 10.3390/biomimetics9070439 (PMC11274816; doi:10.3390/biomimetics9070439)
Supplement: Supplementary file 1 [file biomimetics-09-00439-s001.zip › S1_Milk_carton.pdf]

biomimetics

Supplementary Material S1

Basics of Evolution Strategies: Implementation of the Biomimetic Optimization Method in Educational Modules

Olga Speck [1,2,*], Thomas Speck [1,2], Sabine Baur [2] and Michael Herdy [3]

[1] Cluster of Excellence *liv*MatS @ FIT – Freiburg Center for Interactive Materials and Bioinspired Technologies, 79110 Freiburg, Germany
[2] Plant Biomechanics Group @ Botanic Garden Freiburg, University of Freiburg, 79104 Freiburg, Germany
[3] Ingenieurbüro Herdy (IBH), Kaiserdamm 4, 14057 Berlin, Germany
* Correspondence: olga.speck@biologie.uni-freiburg.de

Educational Module: "Optimization of a Milk Carton"

The presented module is based on scientific research by the Plant Biomechanics Group of the University of Freiburg, Germany [1] in cooperation with INPRO Berlin [2]. Since we use a (1,9)–ES, the educational module is suitable for 9 teams, each with 1 to a maximum of 4 team members from the age of 15 years onward. In addition, 1 dice, 1 calculator, and 2–3 copies of the template "(1,9)–ES Optimization of a milk carton" are required per team. The degree of difficulty is "medium". The experiment can be carried out within 90 minutes.

The following instructions are addressed directly to the students. They are divided into five parts:

(1) Evolution Strategy: General information
(2) Information: Optimized material consumption of a milk carton
(3) Experiment: Experimental setup and performance of the experiment
(4) Evaluation: Analysis of the data
(5) Solutions: Answers or individual solutions to all tasks and a discussion of the experimental results

References

[1] Sauer, S. *Technische Optimierungsverfahren nach dem Vorbild der Natur*; 2009. Unpublished Staatsexamen thesis, University of Freiburg, Germany (in German).
[2] Sauer, S.; Herdy, M.; Speck, T.; Speck, O. Evolutionsstrategie: Optimieren nach dem Vorbild der Natur – Interdisziplinäre Arbeitsweise der Biomechanik und Bionik. *Praxis der Naturwissenschaften – Biologie in der Schule* **2010**, 59, 34–41. (in German).

Publisher's Note: MDPI stays neutral with regard to jurisdictional claims in published maps and institutional affiliations.

Copyright: © 2024 by the authors. Submitted for possible open access publication under the terms and conditions of the Creative Commons Attribution (CC BY) license (https://creativecommons.org/licenses/by/4.0/).

Evolution Strategy

Biomimetic optimization—Is it possible to find the optimal solution without knowing the target? Yes, with the help of Evolution Strategies that are inspired by Darwinian evolution.

Optimization in living nature—Plants and animals are highly adapted to their respective habitats. This is the result of biological evolution that constantly varies the underlying genetic information through an interplay of mutation and recombination and retains individuals with higher reproductive success (= higher fitness) through subsequent selection.

Optimization in technology—Humankind has always striven to improve objects or processes and to find the best solution for given problems: the optimal solution (Fig. S1). Mathematics has its own sub-discipline dedicated to the development of algorithms for solving such optimization problems. In addition to these mathematical optimization methods, some methods are based on biological optimization principles.

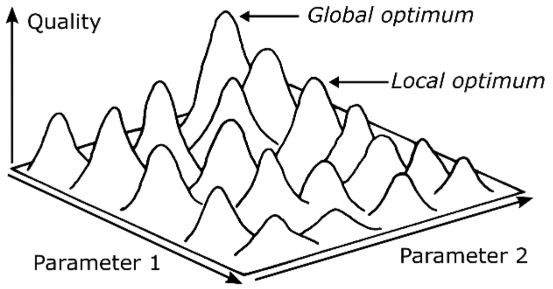

Figure S1: Evolution Strategy in a three-dimensional quality landscape of a maximization problem with two parameters. The landscape exhibits one global maximum and several local maxima.

Evolution Strategy—Evolution Strategy, developed in the 1960s by Ingo Rechenberg and Hans-Paul Schwefel, represents the transfer of the optimization method of biological evolution to technology. It can also be used to solve optimization problems when mathematical solution methods fail. The basic idea of the Evolution Strategy is to change proposed solutions to a formulated optimization problem by random processes (cf. biological mutation) and to combine them with each other (cf. biological recombination) until the optimal solution is found. Following the biological model, proposed solutions used to generate new solutions by mutation and/or recombination are called parent individuals, and the resulting solutions are called offspring individuals. Just as individuals in biology are better or less-well adapted to their environment, some individuals in technology satisfy an optimization criterion better than others, for example,

because they are faster, cheaper, or use less material than their competitors. Whereas conventional optimization methods fail as soon as the quality of an individual cannot be calculated by an appropriate function, the Evolution Strategy can also be applied to problems in which the quality can only be determined experimentally. Analogous to biological selection, the less-efficient solutions are discarded, and only the best solutions are retained.

Variants of Evolution Strategy—Depending on whether the parent individuals participate in the selection process or die beforehand, i.e., are removed from the further optimization process, the following variants of Evolution Strategy can be distinguished:

- **(μ + λ) – ES** (pronounced: mu plus lambda membered evolution strategy): With plus selection, the μ parents are added together with the λ offspring to the ballot box. Therefore, parents and offspring are included in the selection.
- **(μ, λ) – ES** (pronounced: mu comma lambda membered evolution strategy): With comma selection, the μ parents have a limited lifespan and are not added to the λ offspring in the ballot box. Therefore, the patents are not included in the selection.

Evolution window—Evolution, whether biological or artificial, can only take place within the evolution window (Fig. S2). For Evolution Strategy, this means that the individual changes caused by mutation must be neither too small nor too large. Therefore, the "mutation step size" must be chosen optimally. The closer you approach to the optimum, the smaller the steps you should take so as to avoid missing or skipping the optimum. Under certain circumstances, the step size must be refined during an optimization run. This is called “mutative step size control”.

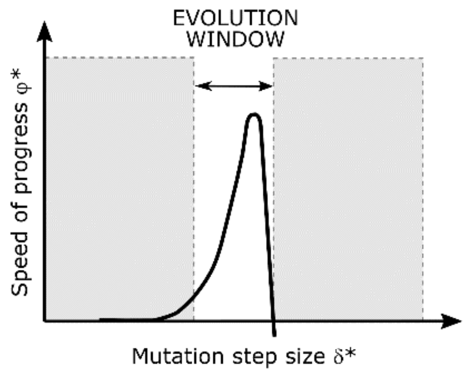

Figure S2: Only if the specific mutation step size δ^* is chosen optimally is there a realistic chance of finding the optimum within a reasonable time. If the mutation step size is too small, stagnation occurs because the specific speed of progress φ^* is close to zero. If the mutation step size is too large, regression may occur because the speed of progress becomes negative.

Optimization of a Milk Carton

One liter of milk needs to be packaged. This can be achieved in many ways but can it be done with a minimum of packaging material? In the experiment below, you can check whether standard milk cartons are optimal in this respect.

The object to be optimized—The primary optimization goal is to minimize the material consumption of the milk carton while maintaining the same volume. If gluing areas and folds are not taken into account, this means that the surface area of the milk carton should be minimized.

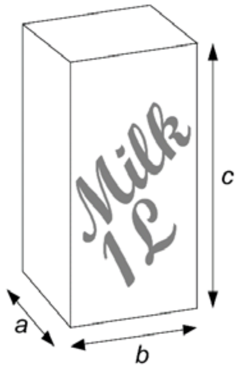

Figure S1.1: Standard cuboid milk carton
Content: 1000 ml
Side length: a = 5 cm, b = 10 cm, c = 20 cm

Optimization—The optimization described in the experimental section uses a (1,9)-membered Evolution Strategy with mutative step size control. In this variant of the Evolution Strategy, each individual consists of *n* object parameter values and a mutation step size. To produce an offspring, the parental step size must always be mutated first in order to enable the parameter values of the parental object to be varied with the resulting offspring step size:

$\delta_O = \delta_P \cdot \xi$ (1) Mutation of the step size

$x_{O_1} = x_{P_1} + \delta_O \cdot z_1, \dots . x_O = x_{P_n} + \delta_O \cdot z_n$ (2) Mutation of object parameters

Whereby the following applies:

δ_P	mutation step size of the parent
δ_O	mutation step size of the offspring
ξ	variation of the mutation step size
$x_{P_1} \dots x_{P_n}$	object parameter of the parent
$x_{O_1} \dots x_{O_n}$	object parameter of the offspring
$z\ (i = 1, \dots n)$	random factor to change the parental object parameter x_i

With mutative step size control, reduced and increased step sizes can be tried in equal proportions in the offspring population. However, the parental step size must not be varied at random. Experts in the field of Evolution Strategy have found that optimization works best when the step size δ is multiplied by a factor of 1.3 in the case of an increase and divided by a factor of 1.3 in the case of a decrease. The mutation step size remains unchanged if $\xi = 1$. The meaning of this in concrete terms is illustrated by the example of a (1,9)-ES with mutative step-size control in Figure S1.2:

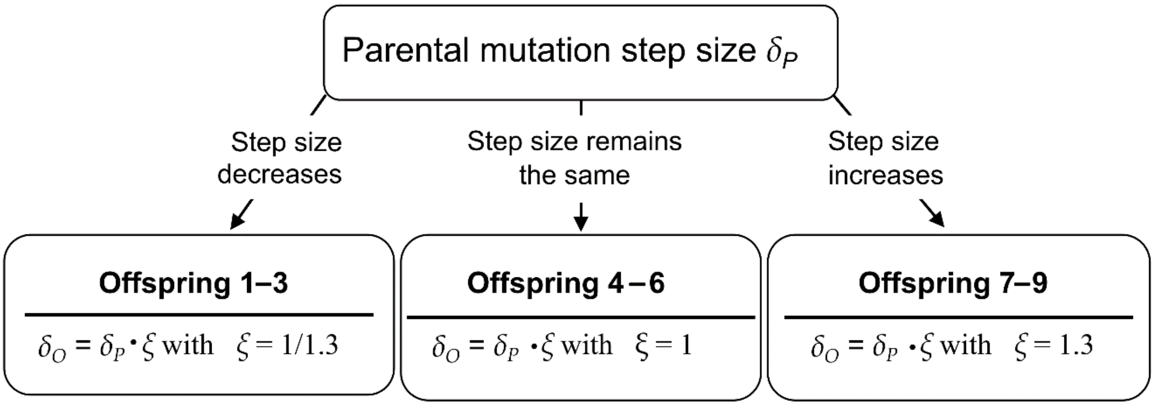

Figure S1.2: Mutative step size control. Since the mutation step size of the offspring is calculated as $\delta_O = \delta_P \cdot \xi$, the mutation step size δ_O decreases if $\xi < 1$, increases if $\xi > 1$, and remains unchanged if $\xi = 1$.

In an Evolution Strategy with mutative step size control, the fact that both smaller and larger step sizes are tried increases the probability that the evolution window will be reached with one of these step sizes (cf. Fig. S2 in "Information ES"). With mutative step size control, we can expect that increasingly those offspring will provide the best quality values and become, in the next generation, parents whose step size is best adapted to the topology of the quality landscape (cf. Fig. S1 in "Information ES"). In other words, the closer you get to the optimum, the more likely it is that offspring with a step size that is too large will miss the optimum and thus produce lower quality values. Conversely, the closer you get to the optimum, the smaller the selected mutation step size will be to avoid overshooting the target and ending up on the way back down

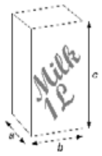

Optimization of a Milk Carton

Optimization with a dice and a pocket calculator—is this possible? Using Evolution Strategy, you can, for example, optimize to a minimum the amount of material needed to package 1 liter of milk.

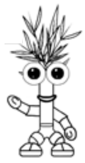	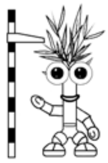	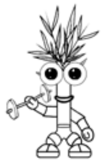	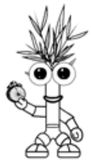
**Working type:**<br>9 teams of equal size<br>(e.g., 1-4 team members)	**Age:**<br>students older than<br>15 years	**Degree of difficulty:**<br>medium	**Duration:**<br>up to 90 minutes

Preparation for the experiment—Answer the following questions and then verify your answers before starting the experiment.

Task 1: What optimization should be performed on the "milk carton"?

___

Task 2: How can the object be efficiently evaluated?

___

Task 3: How many variables does the optimization problem have?

___

Task 4: What constraints limit the optimization?

___

Task 5: If all three variables were changed, what would happen?

___

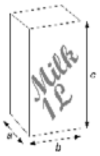

Optimization of a Milk Carton

Instructions for the experiment—Your challenge is to solve the milk carton optimization problem by using a (1,9)-membered Evolution Strategy with mutative step size control (cf. “Information ES”). Divide the class into nine teams of equal size, with each team producing one of the nine offspring. Each team takes a dice, a calculator, and 2-3 copies of the “Template: (1,9)-ES optimization of a milk carton".

1. Each team produces the same offspring in each generation, e.g., always offspring # 8. The corresponding ξ for varying the parental mutation step size is shown in Fig. S1.2 of the information sheet. Each team notes its offspring number and the corresponding ξ in the top right corner of the Template.
2. The values of the standard cuboid milk carton (= starting parent = generation 0) are transferred to the first box of the Template (a_P = 5 cm, b_P = 10 cm, c_P = 20 cm). Since this is an Evolution Strategy with mutative step size control, a start mutative step size (in our case $\delta_P = 0.6$) must be specified in addition to the object parameters (in our case, these are the two side lengths a and b).

Parent of ___1.___ generation	
a_P =	5 cm
b_P =	10 cm
δ_P =	0.6
Quality Q_P =	700 cm²

3. The step size of the offspring δ_O is calculated as indicated in the Template.
4. Each teams throws the dice twice. The number of the first throw generates the random number z_a for the variation of the parental side length a_P, and the number of the second throw generates the random number z_b for the variation of the side length b_P. Note the legend at the top right of the Template for this step. It ensures that the side lengths can become shorter or longer with equal probability. For example, if the first throw was a 5, enter –2 instead of 5 for z_a.
5. The side lengths of the offspring a_O, b_O, and c_O are calculated according to the instructions on the Template.
6. The quality of the offspring Q_O is calculated according to the equation on the Template.
7. From all nine offspring, the one with the smallest Q-value, and therefore the smallest surface area, is selected. This offspring becomes the parent of all teams, and its values are transferred into the next "parent box".
8. Steps 3 to 7 are repeated until the quality of the selected offspring does not change by more than 0.05 cm² for three consecutive generations. Then, after 9 generations at the most, the optimization is finished, even if the condition is not yet met.

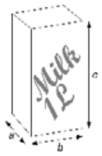

Optimization of a Milk Carton

Task 6: Please record, in the table below, the values of the offspring that you have identified as the best (= lowest value for quality Q) from all teams.

**Generation #**	**Side length a [cm]**	**Side length b [cm]**	**Side length c [cm]**	**Quality Q [cm²]**	**Mutation step size δ [/]**
0	*5.00*	*10.00*	*20.00*	*700.00*	*0.60*
1					
2					
3					
4					
5					
6					
7					
8					
9					

Template: (1,9)-ES Optimization of a milk carton

Parent of_____ generation	Offspring #_ of this generation
a_P =	ξ =
b_P =	
δ_P =	
Quality Q_P =	

dots	⚀	⚁	⚂	⚃	⚄	⚅
z =	1	2	3	-1	-2	-3

Offspring

Strategy parameter:	Mutation:	Evaluation:
$\delta_O = \delta_P \cdot \xi$ =	$a_O = a_P + \delta_O \cdot z_a$ =	$Q_O = 2(ab + ac + bc)$
z_a =	$b_O = b_P + \delta_O \cdot z_b$ =	
z_b =	=> $c_O = 1000/(a \cdot b)$ =	

Parent of_____ generation	Offspring #_ of this generation
a_P =	ξ =
b_P =	
δ_P =	
Quality Q_P =	

dots	⚀	⚁	⚂	⚃	⚄	⚅
z =	1	2	3	-1	-2	-3

Offspring

Strategy parameter:	Mutation:	Evaluation:
$\delta_O = \delta_P \cdot \xi$ =	$a_O = a_P + \delta_O \cdot z_a$ =	$Q_O = 2(ab + ac + bc)$
z_a =	$b_O = b_P + \delta_O \cdot z_b$ =	
z_b =	=> $c_O = 1000/(a \cdot b)$ =	

Parent of_____ generation	Offspring #_ of this generation
a_P =	ξ =
b_P =	
δ_P =	
Quality Q_P =	

dots	⚀	⚁	⚂	⚃	⚄	⚅
z =	1	2	3	-1	-2	-3

Offspring

Strategy parameter:	Mutation:	Evaluation:
$\delta_O = \delta_P \cdot \xi$ =	$a_O = a_P + \delta_O \cdot z_a$ =	$Q_O = 2(ab + ac + bc)$
z_a =	$b_O = b_P + \delta_O \cdot z_b$ =	
z_b =	=> $c_O = 1000/(a \cdot b)$ =	

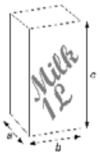

Optimization of a Milk Carton

Task 6: Please record, in the table below, the values of the offspring that you have identified as the best (= lowest value for quality Q) from <u>all</u> teams.

Generation #	Side length a [cm]	Side length b [cm]	Side length c [cm]	Quality Q [cm²]	Mutation step size δ [/]
0	*5*	*10*	*20*	*700*	*0.6*
1					
2					
3					
4					
5					
6					
7					
8					
9					
10					
11					
12					

Task 7: Identify the three-dimensional geometry and side-length dimensions of the material-minimized milk carton.

____

Task 8: Explain why this type of packaging is not used in the marketplace.

____

____

____

Task 9: Use a spreadsheet editor to represent graphically the way in which quality Q and mutation step size δ change during the optimization run. Interpret the graphs.

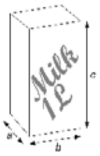

Optimization of a Milk Carton

Preparation for the experiment—Answer the following questions and then verify your answers before starting the experiment.

Task 1: What optimization should be performed on the "milk carton"?
Material consumption should be minimized while maintaining the same volume.

Task 2: How can the object be efficiently evaluated?
The quality can be calculated as follows: $Q(a, b, c) = 2\,(a \cdot b + a \cdot c + b \cdot c)$

Task 3: How many variables does the optimization problem have?
The optimization has two variables.

Task 4: What constraints limit the optimization?
Two constraints limit the optimization: $a, b, c > 0$ *and* $a \cdot b \cdot c = 1000\,ml$

Task 5: If all three variables were changed, what would happen?
If all three variables were changed, some offspring would always arise that violate the constraint $a \cdot b \cdot c = 1000\,ml$*.*

Information ES
Information
Experiment
Evaluation
Solutions

Optimization of a Milk Carton

Task 6: Please record, in the table below, the values of the offspring that you have identified as the best (= lowest value for quality Q) from all teams. (individual solution)

**Generation #**	**Side length a [cm]**	**Side length b [cm]**	**Side length c [cm]**	**Quality Q [cm²]**	**Mutation step size δ [/]**
0	*5.00*	*10.00*	*20.00*	*700.00*	*0.60*
1	5.60	11.20	15.94	661.00	0.60
2	7.40	9.40	14.38	622.00	0.60
3	9.74	10.18	10.09	600.29	0.78
4	10.30	9.58	10.13	600.29	0.60
5	9.84	10.04	10.12	600.04	0.46
6	9.49	10.39	10.14	600.37	0.35
7	10.03	10.12	10.06	600.04	0.27
8	9.63	10.32	10.06	600.24	0.20
9	9.93	10.17	9.90	600.04	0.15

Task 7: Identify the three-dimensional geometry and side-length dimensions of the material-minimized milk carton.
The material-optimized milk carton is a cube with side lengths of 10 cm.

Task 8: Explain why this type of packaging is not used in the marketplace.
This type of packaging is probably not used in the marketplace because beverage packaging manufacturers have to consider aspects other than minimal material use, such as ease of handling, resealability, production-related adhesive edges and folds, and the size of the advertising space. Moreover, few people would buy a milk carton that does not fit into the refrigerator door with a depth of approx. 7 cm.

Task 9: Use a spreadsheet editor to represent graphically the way that quality Q and mutation step size δ change during the optimization run. Interpret the graphs.

In principle, two graphs can be generated: (1) the quality Q (y-axis) as a function of the number of generations (x-axis) and (2) the mutation step size δ as a function of the number of generations (x-axis). Figure S1.3 shows these two graphs combined by using two y-axes.

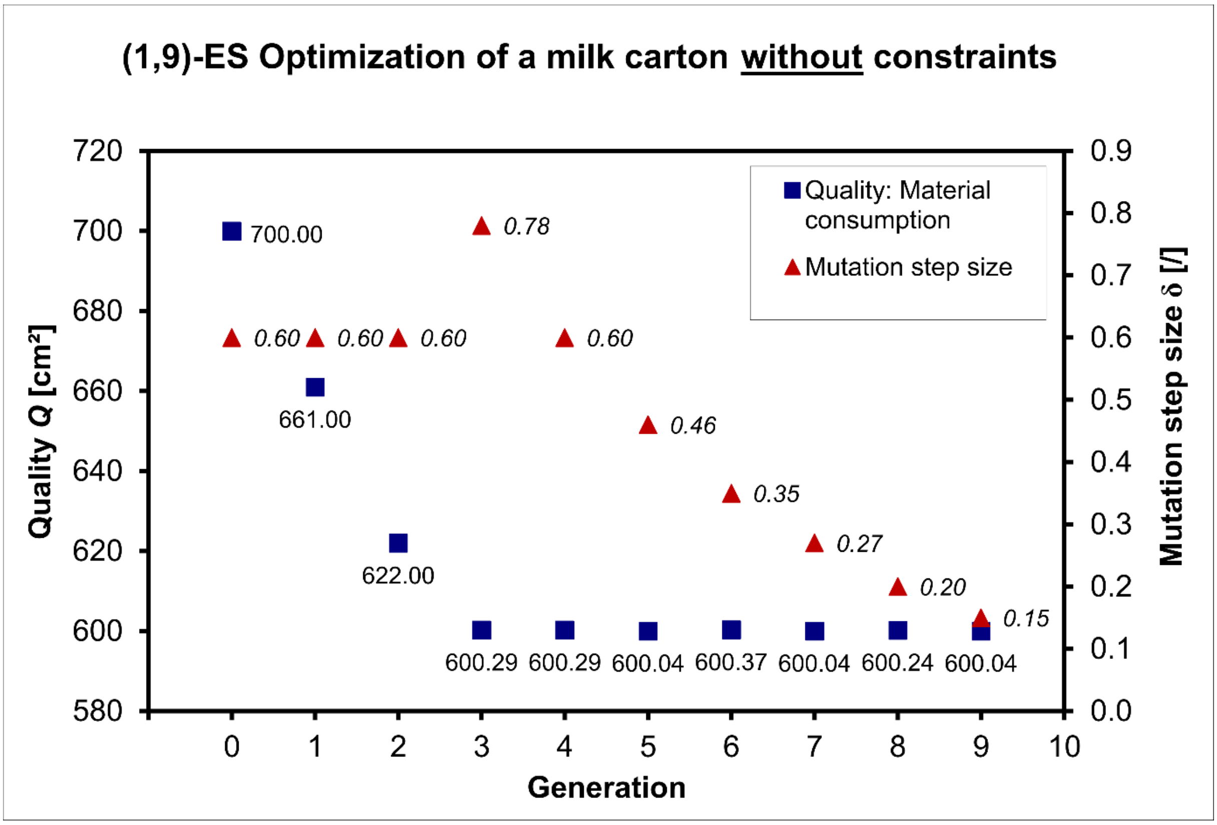

Figure S1.3: Optimization of the material consumption of a milk carton with a (1,9)-membered Evolution Strategy (individual solution). The graph combines the quality Q and the mutation step size δ as a function of the number of generations. The quality decreases rapidly and, starting with the 3rd generation, levels off at the optimum value of 600 cm². The step size of the mutation also decreases as the number of generations increases. Note: In both cases, the data points must not be connected to form a curve, as there is no generation 5.3, for example.

Interpretation of the graph showing the quality Q as a function of the number of generations: *We observe that a quality value close to the optimum is reached relatively quickly. In subsequent generations, the quality slowly but surely levels off at the optimum value of 600 cm². A temporary deterioration of the selected quality value can be explained because, in the respective generation, all offspring had higher Q values and thus higher material consumption than the parent, and because the parent does not participate in the selection process as a result of the comma selection.*

Interpretation of the graph showing the mutation step size δ as a function of the number of generations: *The mutation step size decreases as the number of generations increases. This is because offspring generated with a large step size will only ensure rapid progress toward the optimum at the beginning of the optimization. The closer you approach the optimum value, the more likely it is that offspring with a step size that is too large will miss the optimum and therefore deliver poorer quality values. In other words, the closer you approach the optimum value, the smaller the selected mutation step size becomes.*
