# Supplementary material for: The Basics of Evolution Strategies: The Implementation of the Biomimetic Optimization Method in Educational Modules
Source: Biomimetics (Basel). 2024 Jul 18;9(7):439. doi: 10.3390/biomimetics9070439 (PMC11274816; doi:10.3390/biomimetics9070439)
Supplement: Supplementary file 1 [file biomimetics-09-00439-s001.zip › S2_Brachistochrone.pdf]

biomimetics

MDPI

Supplementary Material S2

Basics of Evolution Strategies: Implementation of the Biomimetic Optimization Method in Educational Modules

Olga Speck [1,2,*], Thomas Speck [1,2], Sabine Baur [2] and Michael Herdy [3]

1 Cluster of Excellence *liv*MatS @ FIT – Freiburg Center for Interactive Materials and Bioinspired Technologies, 79110 Freiburg, Germany
2 Plant Biomechanics Group @ Botanic Garden Freiburg, University of Freiburg, 79104 Freiburg, Germany
3 Ingenieurbüro Herdy (IBH), Kaiserdamm 4, 14057 Berlin, Germany
* Correspondence: olga.speck@biologie.uni-freiburg.de

Educational Module: "Fastest and Shortest Marble Track"

The presented module is based on scientific research by the Plant Biomechanics Group of the University of Freiburg, Germany [1] in cooperation with INPRO Berlin [2]. We present the construction plans of a marble track with a fixed straight line and a fixed brachistochrone curve, in which the difference between the running times of the shortest and the fastest track can be measured and even seen with the naked eye.

Using the work sheets in the educational module, students will learn the physical principles that explain why the shortest path is not the fastest path. The module requires students to be familiar with mathematical equations and to understand the theorem of rays. The marble track can be built by teams of at least 2 members from the age of 15 years onward. The degree of difficulty is "medium". The low-cost construction can be completed within 2 x 90 minutes. It takes another 90 minutes to perform the experiments, evaluate the results, and answer the questions.

The following instructions are addressed directly to the students. They are divided into five parts:

(1) Evolution Strategy: General information
(2) Information: The brachistochrone problem
(3) Experiment: Construction of a marble track, experimental setup, and experiments
(4) Evaluation: Analysis of the data
(5) Solutions: Answers or individual solutions to all tasks and a discussion of the experimental results

Publisher's Note: MDPI stays neutral with regard to jurisdictional claims in published maps and institutional affiliations.

Copyright: © 2024 by the authors. Submitted for possible open access publication under the terms and conditions of the Creative Commons Attribution (CC BY) license (https://creativecommons.org/licenses/by/4.0/).

References

[1] Sauer, S. *Technische Optimierungsverfahren nach dem Vorbild der Natur*; 2009. Unpublished Staatsexamen thesis, University of Freiburg, Germany (in German).

[2] Sauer, S.; Herdy, M.; Speck, T.; Speck, O. Evolutionsstrategie: Optimieren nach dem Vorbild der Natur – Interdisziplinäre Arbeitsweise der Biomechanik und Bionik. *Praxis der Naturwissenschaften – Biologie in der Schule* **2010**, 59, 34–41. (in German).

Evolution Strategy

Biomimetic optimization—Is it possible to find the optimal solution without knowing the target? Yes, with the help of Evolution Strategies that are inspired by Darwinian evolution.

Optimization in living nature—Plants and animals are highly adapted to their respective habitats. This is the result of biological evolution that constantly varies the underlying genetic information through an interplay of mutation and recombination and retains individuals with higher reproductive success (= higher fitness) through subsequent selection.

Optimization in technology—Humankind has always striven to improve objects or processes and to find the best solution for given problems: the optimal solution (Fig. S1). Mathematics has its own sub-discipline dedicated to the development of algorithms for solving such optimization problems. In addition to these mathematical optimization methods, some methods are based on biological optimization principles.

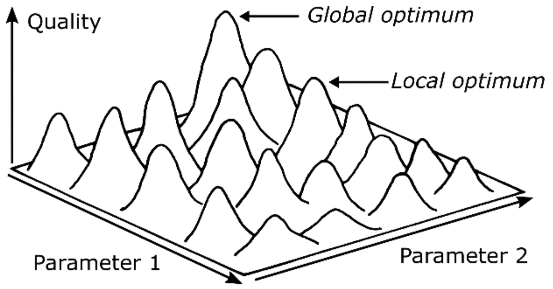

Figure S1: Evolution Strategy in a three-dimensional quality landscape of a maximization problem with two parameters. The landscape exhibits one global maximum and several local maxima.

Evolution Strategy—Evolution Strategy, developed in the 1960s by Ingo Rechenberg and Hans-Paul Schwefel, represents the transfer of the optimization method of biological evolution to technology. It can also be used to solve optimization problems when mathematical solution methods fail. The basic idea of the Evolution Strategy is to change proposed solutions to a formulated optimization problem by random processes (cf. biological mutation) and to combine them with each other (cf. biological recombination) until the optimal solution is found. Following the biological model, proposed solutions used to generate new solutions by mutation and/or recombination are called parent individuals, and the resulting solutions are called offspring individuals. Just as individuals in biology are better or less-well adapted to their environment, some individuals in technology satisfy an optimization criterion better than others, for example,

because they are faster, cheaper, or use less material than their competitors. Whereas conventional optimization methods fail as soon as the quality of an individual cannot be calculated by an appropriate function, the Evolution Strategy can also be applied to problems in which the quality can only be determined experimentally. Analogous to biological selection, the less-efficient solutions are discarded, and only the best solutions are retained.

Variants of Evolution Strategy—Depending on whether the parent individuals participate in the selection process or die beforehand, i.e., are removed from the further optimization process, the following variants of Evolution Strategy can be distinguished:

- **(μ + λ) – ES** (pronounced: mu plus lambda membered evolution strategy): With plus selection, the μ parents are added together with the λ offspring to the ballot box. Therefore, parents and offspring are included in the selection.
- **(μ, λ) – ES** (pronounced: mu comma lambda membered evolution strategy): With comma selection, the μ parents have a limited lifespan and are not added to the λ offspring in the ballot box. Therefore, the patents are not included in the selection.

Evolution window—Evolution, whether biological or artificial, can only take place within the evolution window (Fig. S2). For Evolution Strategy, this means that the individual changes caused by mutation must be neither too small nor too large. Therefore, the "mutation step size" must be chosen optimally. The closer you approach to the optimum, the smaller the steps you should take so as to avoid missing or skipping the optimum. Under certain circumstances, the step size must be refined during an optimization run. This is called “mutative step size control”.

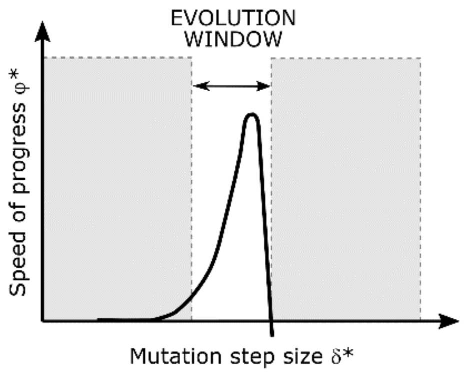

Figure S2: Only if the specific mutation step size δ^* is chosen optimally is there a realistic chance of finding the optimum within a reasonable time. If the mutation step size is too small, stagnation occurs because the specific speed of progress φ^* is close to zero. If the mutation step size is too large, regression may occur because the speed of progress becomes negative.

The Brachistochrone Problem

Is the shortest path also the fastest? Of all the curves, the straight line is undoubtedly the shortest geometrically. But does it also represent the one that is the shortest in time? This question can be answered either purely mathematically or with the help of Evolution Strategy, which, as in biological evolution, optimizes by trial and error.

A competition from 1696—The Swiss mathematician Johann Bernoulli was thinking about this question as early as 1696, when he posed the so-called brachistochrone problem originally in Latin to his fellow mathematicians: “Given two points A and B in a vertical plane, what is the curve traced out by a point acted on only by gravity, which starts at A and reaches B in the shortest time?'' [1]. Five solutions to Johann Bernoulli's problem were published by himself and other scientists in January and May 1697. The optimal trajectory is also known as the "fastest path" or "brachistochrone" (Greek: brachýs "short", chrónos "time").

The fastest and the shortest path—There are several ways to connect point A and point B, which are not at the same height and not directly below each other (Fig. S2.1). Both the shortest path (= a straight line) and the fastest path (= the brachistochrone curve) are optimization problems.

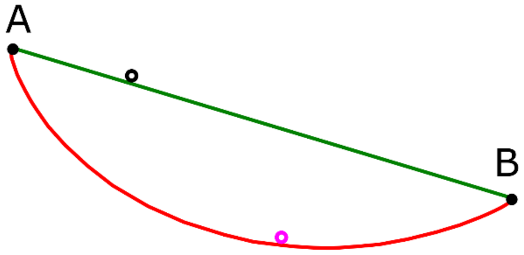

Figure S2.1: The points A and B can be connected by the shortest path (= a straight line, in green) and the fastest path (= the brachistochrone curve, in red). The black and magenta marbles start together at A but have traveled different distances by B.

The brachistochrone curve—Johann Bernoulli himself and some of his colleagues discovered that the brachistochrone has the shape of a cycloid. Such a cycloid is obtained by rolling a circle on a straight line and observing a fixed point on the edge of the circle (Fig. S2.2).

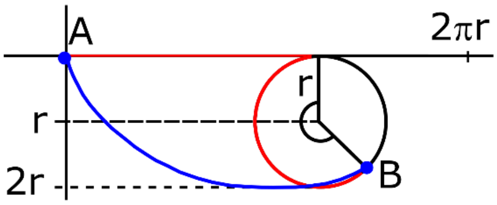

Figure S2.2: A cycloid is created by a rolling circle. If a *circle with a radius r rolls along a straight line, a fixed point on the edge of the circle describes a cycloid (blue) with a period of $2\pi r$ (= circumference).*

The Brachistochrone Problem

For all points on the cycloid, a marble starting at A will reach its destination fastest if it rolls along the cycloid. The really amazing thing is that the cycloid is the shortest path, even if A and B are so far apart that the marble has to roll slightly uphill!

Construction of the brachistochrone between given points—Given two points A and B to which you want to find the fastest path, you know that it has the shape of a cycloid, but to draw the corresponding cycloid segment, you need to know the radius of the corresponding circle. The radius can be easily determined by following the steps below:

1. The two given points A and B are connected by the line AB (Fig. S2.3).
2. Describe an arbitrary cycloid under the horizontal line through A, which only has to start at A. This cycloid will intersect the line AB at point R and the horizontal line at point S (Fig. S2.3).

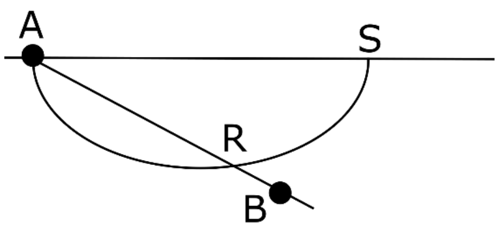

Figure S2.3: Construction steps 1 and 2 include the description of an arbitrary cycloid that intersects the line AB at point R.

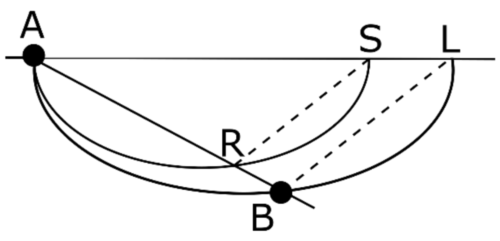

Figure S2.4: Construction step 3 to find the fastest path between A and B by centric stretching so that point R is mapped onto the point B.

3. The triangle ARS is now stretched by centric stretching so that the point R is mapped onto the point B. Since the image lines are always parallel to the original image line during centric stretching, the image line to RS can easily be drawn. It intersects the horizontal line at point L (Fig. S2.4).
4. With the 1st theorem of rays and the periodicity of the cycloids, it now follows that:

$$\frac{AB}{AR} = \frac{AL}{AS} = \frac{2\pi \cdot r_{ABL}}{2\pi \cdot r_{ARS}} = \frac{r_{ABL}}{r_{ARS}} \qquad (1)$$

   In equation (1) r_{ARS} is the circle radius of the given cycloid, and r_{ABL} is the circle radius of the cycloid being sought.

5. Solving the formula for r_{ABL} gives: $r_{ABL} = \frac{AB}{AR} \cdot r_{ARS}$ (2)
6. All the variables needed to calculate r_{ABL} are known, so the cycloid connecting points A and B can now be constructed easily.

[1] Bernoulli, J. Problema novum ad cujus solutionem Mathematici invitantur. *Acta Eruditorum* **1696**, 18.

Fastest and Shortest Marble Track

Have you ever thought of an application in engineering in which it would be better to use a brachistochrone curve instead of a straight line? For example, when rescuing people from an airplane, speed is of the essence, so an emergency slide should have a brachistochrone curve.

Experiments—If you want to take a closer look at the brachistochrone problem, you can either use the marble track experimental setup described below to compare the straight line as the shortest path and the cycloid (= brachistochrone curve) as the fastest path or you can perform experiments simulating various marble track shapes by using the software “EvoBrach” (cf. Supplementary Materials Files S3 and S4).

Construction of a low-cost marble track

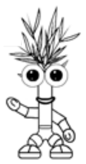	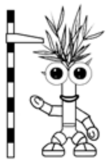	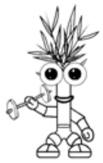	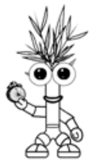
**Working type:** at least 1 team of 2 team members	**Age:** students older than 15 years	**Degree of difficulty:** medium	**Duration:** 3 x 90 minutes

Materials required to build the low-cost marble track

Amount	Material	Dimensions	Supply source
2	transparent plastic tubes (garden hose), inner diameter ≈ 16 mm	each 2.30 m long	hardware store
1	wooden slat	ca. 2.4 cm x 4.8 cm x 200 cm	hardware store
20	hose clamps	diameter: at least 12-22 mm	hardware store
≥ 20	cable ties	290 mm long	hardware store
2	plastic flower pots	diameter: ca. 8 cm	hardware store
2	semi-precious stone beads (marbles)	diameter: 10 mm, mass > 1.3 g	hobby supplies
2	cardboard boxes, e.g., moving box	80 cm x 200 cm, 75 cm x 75 cm	—
2	tripod stand	—	school inventory
2	tripod stand	at least 75 cm long	school inventory
2	screw clamps	—	school inventory
2	universal sockets		school inventory
**Other:** Scissors, carpet knife, tape, duct tape, thumbtacks, pencil, permanent marker, meter stick or paper tape measure, cross-tip screwdriver and slotted screwdriver, string (at least 45 cm long), stopwatch.			

Fastest and Shortest Marble Track

Experimental set up

- Draw a circle with a radius of 35 cm on a square piece of cardboard. You can use a pencil, string or tape measure, and a thumbtack to help you.

- Cut out the circle. Mark any point P on the edge of the circle and draw a line connecting point P with the center of the circle.

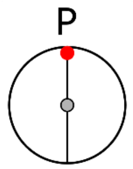

- Place the cardboard on the floor and slide it with its longer side against a wall. Position the circle so that the point P is at the upper left corner of the cardboard, and that the line connecting point P with the center of the circle is perpendicular to the wall.

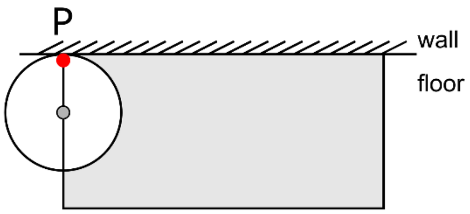

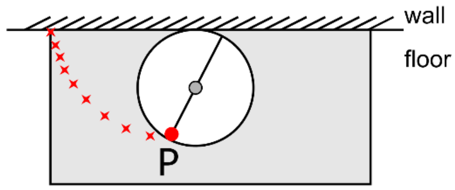

- Roll the circle along the wall to the right. Trace the path that point P describes by drawing a small cross on the surface at regular intervals where point P is currently located.

- Connect the crosses that you have drawn to form a curve (= cycloid). You can see whether you have drawn this correctly if the dimensions of your drawing correspond approximately to the dimensions in the following diagram. If they do not match, please redraw the line. Label the starting point and end point of the marble track with A and B as shown in the figure.

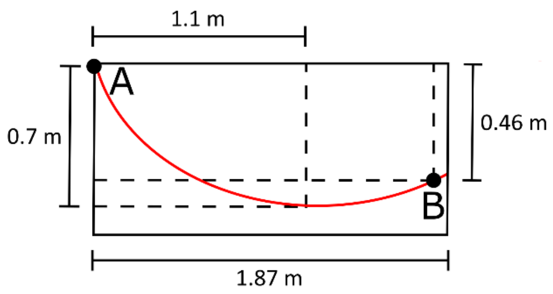

- In the next step, we fix one of the hoses to the cardboard by using cable ties along the pre-drawn curve. To prevent the hose from deforming when the cable ties are attached, at least 6 hose clamps must be attached to the transparent plastic tubes beforehand: one at the starting point A, one just before the end point B, one at the minimum point of the curve, and the rest at regular intervals somewhere in between. To do this, position the transparent plastic tube including the hose clamps on the cardboard. The tube should start directly at point A and continue as follows:

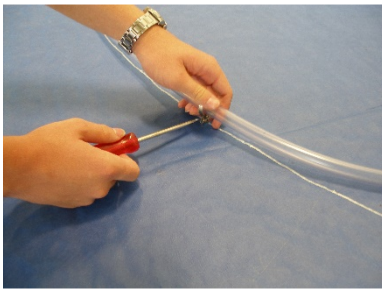

- Carefully tighten the hose clamps.

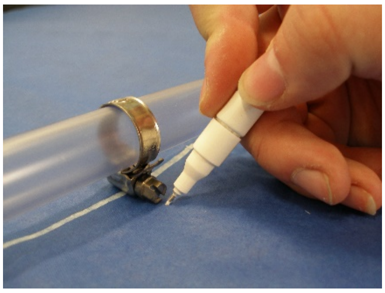

- Mark the edge of the clamp on the left and right.

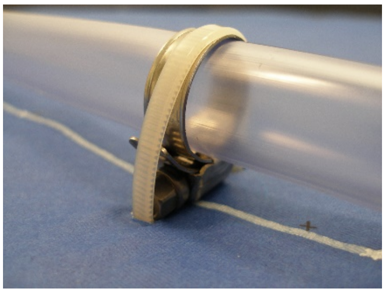

- Drill holes at the marked points, thread in the cable ties through the holes and lash them to the back of the cardboard.

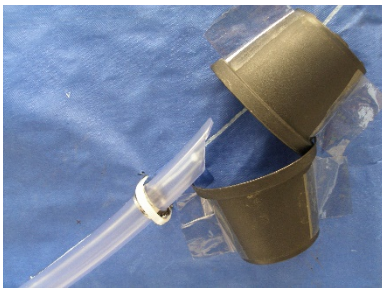

- Cut the tube approx. 2-3 cm behind the end point B. Cut the plastic flower pot in half and attach it as shown.

- Place the entire construction vertically and fix it to the wall or a table in this position.

Fastest and Shortest Marble Track

- The second tube for the straight line is cut approx. 2 cm at the proposed starting point A.
- Attach it to the upright wooden slat by using cable ties and then positioned it so that a marble rolling down it will start at point A and pass point B on its way down.
- The marbles are collected in a plastic pot.

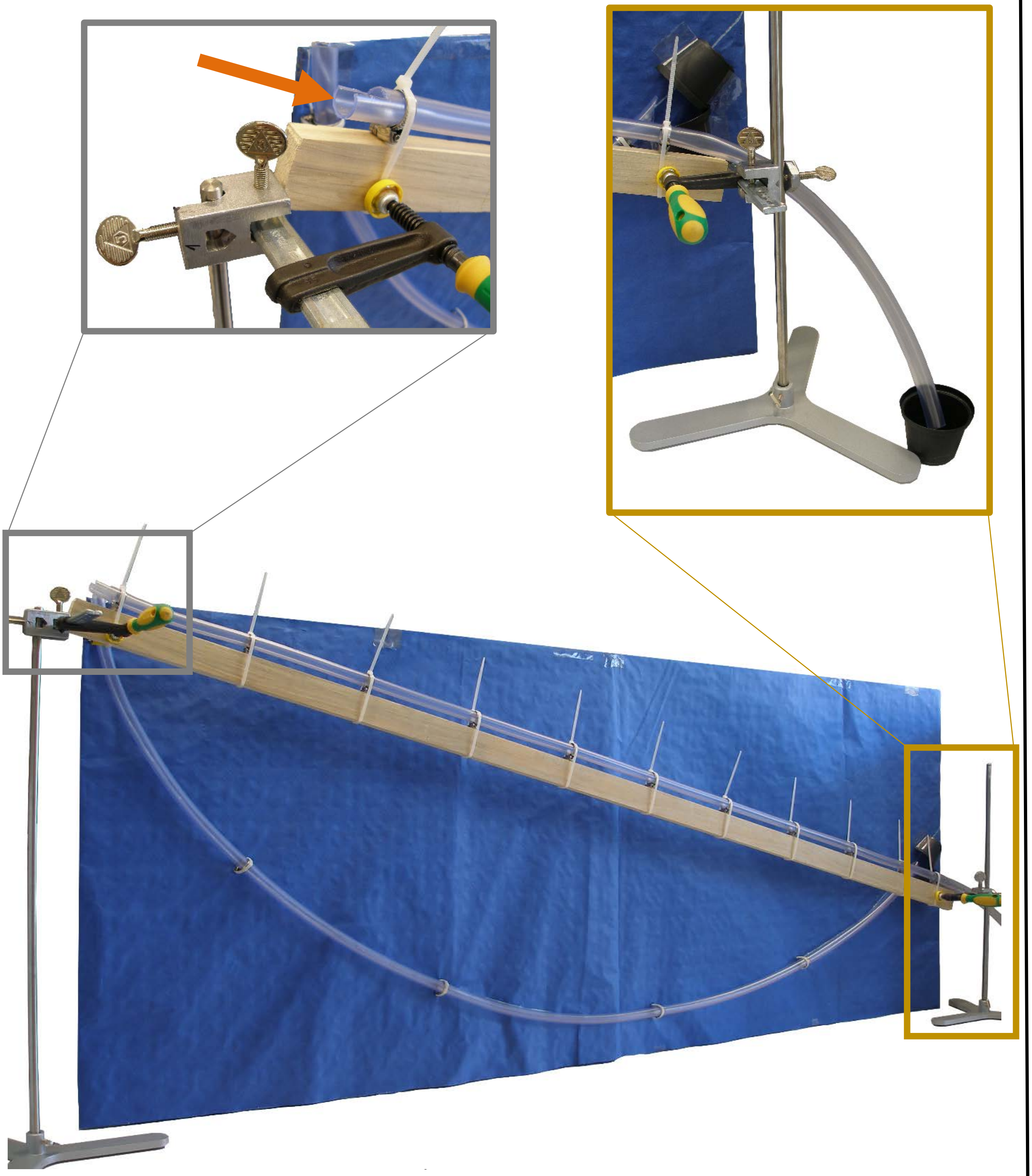

Fastest and Shortest Marble Track

Performing the experiment—You are now ready to determine the running times of the marbles in the experiment. Use a stopwatch to measure the time that it takes the marble to travel from A to B ten times for each track. Record the times in seconds in the table below. Then calculate the average.

Note: The marbles should not gain momentum when they are released. To ensure this on the straight path, you can position the marble and use an obstacle (e.g., screwdriver) to prevent it from rolling away prematurely. At the start, the obstacle is removed, and the marble slides down the track using only gravity. The brachistochrone curve requires your dexterity. Carefully release the marble without giving it extra acceleration.

Task 1: Perform ten experimental measurements on the marble track that you have constructed. Record the running times on the straight line and on the brachistochrone curve. Calculate the average of each of the ten measurements.

**experiment #**	**straight line time [s]**	**brachistochrone curve time [s]**
1		
2		
3		
4		
5		
6		
7		
8		
9		
10		
**Mean**		

Fastest and Shortest Marble Track

Task 1: Perform ten experimental measurements on the marble track that you have constructed. Record the running times on the straight line and on the brachistochrone curve. Calculate the average of each of the ten measurements.

**experiment #**	**straight line time [s]**	**brachistochrone curve time [s]**
1		
2		
3		
4		
5		
6		
7		
8		
9		
10		
**Mean**		

Fastest and Shortest Marble Track

The tasks 2-8 can be solved using the equations and drawings in Box 1-3. Friction is not considered in any case.

Box 1: Running times

$g \approx 9.81$ m/s² (gravitational acceleration)

Straight line:

$$t_S = \sqrt{\frac{2}{g} \cdot \left(\frac{w^2}{h} + h\right)}$$

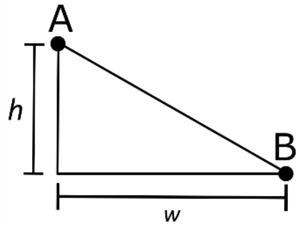

Brachistochrone curve:

$$t_C = 2 \cdot \sqrt{\frac{r}{g}} \cdot \left(\frac{\pi}{2} + tan^{-1}\sqrt{\frac{2r - (h_A - h_B)}{(h_A - h_B)}}\right)$$

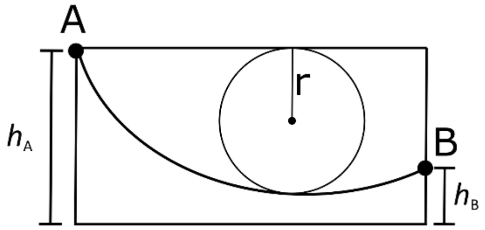

Task 2: Calculate the running times of the marble on the two tracks.

Task 3: Compare theoretical running times with the experimentally determined running times and interpret the results.

Box 2: Velocity

The velocity of a marble at the point D of any curve is a function only of the difference in the height that it travels: $v = \sqrt{2g(h_A - h_D)}$

Task 4: Consider at which position on the straight line the marble reaches its maximum velocity and calculate it (cf. Box 2).

Task 5: Discuss and calculate where on the brachistochrone curve the marble reaches its maximum velocity (cf. Box 2).

Task 6: Calculate the final velocity of the marble at point B for the straight line and for the brachistochrone curve.

Box 3: Law of conservation of energy

potential energy in point A — kinetic energy in point B — potential energy in point B

$$m \cdot g \cdot h_A = \frac{1}{2} m \cdot v^2 + m \cdot g \cdot h_B$$

where m is the mass of the marble, g is the gravitational acceleration (g ≈ 9.81 m/s²), v is the velocity, h_A the height at point A, and h_B the height at point B.

Task 7: Use the law of conservation of energy (cf. Box 3) to discuss your results. Explain why the final velocities of the marble at point B are the same on both paths (cf. task 6), but the running times are different (cf. task 2).

Task 8: Imagine applications for a brachistochrone curve in technology.

Fastest and Shortest Marble Track

Task 1: Perform ten experimental measurements on the marble track that you have constructed. Record the running times on the straight line and on the brachistochrone curve. Calculate the average of each of the ten measurements (individual solution).

experiment #	straight line time [s]	brachistochrone curve time [s]
1	1.34	0.85
2	1.49	1.04
3	1.59	1.03
4	1.35	1.01
5	1.41	1.00
6	1.61	1.07
7	1.29	1.02
8	1.41	1.06
9	1.34	1.01
10	1.57	1.04
**Mean**	1.44	1.013

Task 2: Calculate the running times of the marble on the two tracks.

Straight line: $t_S = \sqrt{\frac{2}{9.81\ \mathrm{m/s^2}} \cdot \left(\frac{1.87^2\ \mathrm{m^2}}{0.46\ \mathrm{m}} + 0.46\ \mathrm{m}\right)} \approx \mathbf{1.282\ s}$

Brachistochrone curve: $t_C = 2 \cdot \sqrt{\frac{0.35\ \mathrm{m}}{9.81\ \mathrm{m/s^2}}} \cdot \left(\frac{\pi}{2} + tan^{-1}\sqrt{\frac{2 \cdot 0.35\ \mathrm{m} - (0.70\ \mathrm{m} - 0.24\ \mathrm{m})}{(0.70\ \mathrm{m} - 0.24\ \mathrm{m})}}\right) \approx \mathbf{0.83\ s}$

Task 3: Compare theoretical running times with the experimentally determined running times and interpret the results.

Experimental determination of running times gives higher values. Possible causes: friction, reaction time when pressing the stopwatch. However, the ratios remain roughly the same.

$$\frac{t_{S\_theoretical}}{t_{C\_theoretical}} \approx \frac{t_{S\_measured}}{t_{C\_measured}}$$

Given the values from task 2, we find a ratio of $1.282\ /\ 0.83\ =\ 1.54$ *for the straight line and* $1.44\ /\ 1.013\ =\ 1.42$ *for the brachistochrone curve.*

Task 4: Consider at which position on the straight line the marble reaches its maximum velocity and calculate it.

The velocity depends only on the difference in height covered. Therefore, the highest velocity is reached at the end point B.

$$v_{S\_max} = \sqrt{2 \cdot g(h_A - h_B)} = \sqrt{2 \cdot 9.81\ \mathrm{m/s^2} \cdot (0.70\ m - 0.24\ m)} \approx \mathbf{3\ m/s}$$

Task 5: Discuss and calculate where on the brachistochrone curve the marble reaches its maximum velocity.

The highest speed is reached at the minimum point of the curve, as this is the point at which the greatest height difference lies.

$$v_{C\_max} = \sqrt{2 \cdot g(h_A - h_{min})} = \sqrt{2 \cdot 9.81\ \mathrm{m/s^2} \cdot (0.70\ \mathrm{m})} \approx \mathbf{3.7\ m/s}$$

Task 6: Calculate the final velocity of the marble at point B for the straight line and for the brachistochrone curve.

At the end point B, both of the marbles have covered a height difference of $0.70\ m - 2.24\ m$*. This means that they both have the same velocity at point B, which is calculated as follows:*

$$v_{S\_B} = v_{C\_B} = \sqrt{2 \cdot g(h_A - h_B)} = \sqrt{2 \cdot 9.81\ \mathrm{m/s^2} \cdot (0.70\ \mathrm{m} - 0.24\ \mathrm{m})} \approx \mathbf{3\ m/s}$$

Task 7: Use the law of conservation of energy to discuss your results. Explain why the final velocities of the marble at point B are the same on both paths (cf. task 6), but the running times are different (cf. task 2).

Since both marbles have the same potential energy at point A and point B, their kinetic energy (≙ velocity) at the end point B must also be the same. As shown in task 6, the velocity is approximately 3 m/s in both cases. On the other hand, the maximum velocities and the running times of the marbles are different for the two path types. Although the brachistochrone curve is much longer than the straight line, it has a steeper slope at the beginning, allowing more potential energy to be converted into kinetic energy. In other words, the marble accelerates faster on the brachistochrone curve and therefore has a higher average velocity.

Task 8: Imagine applications for a brachistochrone curve in technology.

Brachistochrone curves are useful wherever a distance needs or wants to be covered as quickly as possible. Possible applications are the profiles of emergency slides, half-pipes [1], ski jumping hills, pipelines [2], velodromes [3], alpine ski routes for ski racing [4], and starting ramps of rollercoasters and race tracks.

References

[1] Jiang, Y., Liu, C., Ye, Y., Li, J., Dong, Y. On the discrete algorithm of maximum velocity curve about halfpipe. In: 2012 24th *Chinese Control and Decision Conference* (CCDC). IEEE, 2012, 679-681.

[2] Ganesh, M., Sethuraman, N., Rangasamy, V., Navinkumar, S., Dhinagaran, S. High efficiency hydro-powerplant with brachistochrone curve pipe. *International Journal of Science Academic Research* **2022**, 11(3), 4725-4728. Online available at https://www.scienceijsar.com/sites/default/files/article-pdf/IJSAR-1304.pdf

[3] Benham, G.P., Cohen, C., Brunet, E., Clanet, C. Brachistochrone on a velodrome. *Proceedings of the Royal Society A* **2020,** 476(2238), 20200153. DOI: 10.1098/rspa.2020.0153

[4] Nørstrud, H. *Alpine Downhill and Speed-Skiing.* In: Nørstrud, H. (eds) Sport Aerodynamics. CISM International Centre for Mechanical Sciences 2008, 506, 131-138. Springer, Vienna. DOI: 10.1007/978-3-211-89297-8_7
