# Supplementary material for: The Basics of Evolution Strategies: The Implementation of the Biomimetic Optimization Method in Educational Modules
Source: Biomimetics (Basel). 2024 Jul 18;9(7):439. doi: 10.3390/biomimetics9070439 (PMC11274816; doi:10.3390/biomimetics9070439)
Supplement: Supplementary file 1 [file biomimetics-09-00439-s001.zip › S3_EvoBrach.pdf]

biomimetics

MDPI

Supplementary Material S3

Basics of Evolution Strategies: Implementation of the Biomimetic Optimization Method in Educational Modules

Olga Speck [1,2,*], Thomas Speck [1,2], Sabine Baur [2] and Michael Herdy [3]

[1] Cluster of Excellence *liv*MatS @ FIT – Freiburg Center for Interactive Materials and Bioinspired Technologies, 79110 Freiburg, Germany
[2] Plant Biomechanics Group @ Botanic Garden Freiburg, University of Freiburg, 79104 Freiburg, Germany
[3] Ingenieurbüro Herdy (IBH), Kaiserdamm 4, 14057 Berlin, Germany
* Correspondence: olga.speck@biologie.uni-freiburg.de

Educational Module: "Various Marble Track Shapes"

The presented module is based on scientific research by the Plant Biomechanics Group of the University of Freiburg, Germany [1] in cooperation with INPRO Berlin [2]. The software "EvoBrach" is a demonstration program that has been developed for students and that simulates the use of the Evolution Strategy to find the brachistochrone for a given start and ending point configuration. In digital experiments, it can be used to compare the running times of a marble on a straight line, a parabola, and a brachistochrone curve.

The experiments can be performed in teams or individually. The degree of difficulty is "medium". It takes about 90 minutes to perform the digital experiments, evaluate the results, and answer the questions. We provide the EvoBrach.exe file and the German manual in Supplementary Materials File S4.

The following instructions are addressed directly to the students. They are divided into five parts:

(1) Evolution Strategy: General information
(2) Information: The brachistochrone problem
(3) Experiment: Introduction to the software "Evobrach" and digital experiments
(4) Evaluation: Analysis of the data
(5) Solutions: Answers or individual solutions to all tasks and a discussion of the experimental results

Publisher's Note: MDPI stays neutral with regard to jurisdictional claims in published maps and institutional affiliations.

Copyright: © 2024 by the authors. Submitted for possible open access publication under the terms and conditions of the Creative Commons Attribution (CC BY) license (https://creativecommons.org/licenses/by/4.0/).

References

[1] Sauer, S. *Technische Optimierungsverfahren nach dem Vorbild der Natur*; 2009. Unpublished Staatsexamen thesis, University of Freiburg, Germany (in German).
[2] Sauer, S.; Herdy, M.; Speck, T.; Speck, O. Evolutionsstrategie: Optimieren nach dem Vorbild der Natur – Interdisziplinäre Arbeitsweise der Biomechanik und Bionik. *Praxis der Naturwissenschaften – Biologie in der Schule* **2010**, 59, 34–41. (in German).

Evolution Strategy

Biomimetic optimization—Is it possible to find the optimal solution without knowing the target? Yes, with the help of Evolution Strategies that are inspired by Darwinian evolution.

Optimization in living nature—Plants and animals are highly adapted to their respective habitats. This is the result of biological evolution that constantly varies the underlying genetic information through an interplay of mutation and recombination and retains individuals with higher reproductive success (= higher fitness) through subsequent selection.

Optimization in technology—Humankind has always striven to improve objects or processes and to find the best solution for given problems: the optimal solution (Fig. S1). Mathematics has its own sub-discipline dedicated to the development of algorithms for solving such optimization problems. In addition to these mathematical optimization methods, some methods are based on biological optimization principles.

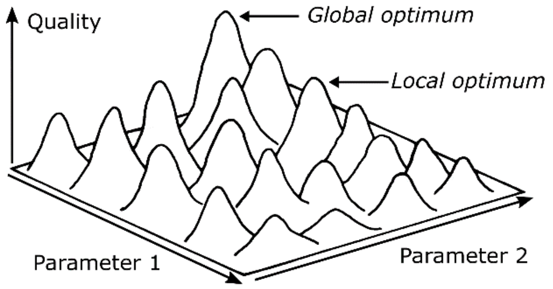

Figure S1: Evolution Strategy in a three-dimensional quality landscape of a maximization problem with two parameters. The landscape exhibits one global maximum and several local maxima.

Evolution Strategy—Evolution Strategy, developed in the 1960s by Ingo Rechenberg and Hans-Paul Schwefel, represents the transfer of the optimization method of biological evolution to technology. It can also be used to solve optimization problems when mathematical solution methods fail. The basic idea of the Evolution Strategy is to change proposed solutions to a formulated optimization problem by random processes (cf. biological mutation) and to combine them with each other (cf. biological recombination) until the optimal solution is found. Following the biological model, proposed solutions used to generate new solutions by mutation and/or recombination are called parent individuals, and the resulting solutions are called offspring individuals. Just as individuals in biology are better or less-well adapted to their environment, some individuals in technology satisfy an optimization criterion better than others, for example,

because they are faster, cheaper, or use less material than their competitors. Whereas conventional optimization methods fail as soon as the quality of an individual cannot be calculated by an appropriate function, the Evolution Strategy can also be applied to problems in which the quality can only be determined experimentally. Analogous to biological selection, the less-efficient solutions are discarded, and only the best solutions are retained.

Variants of Evolution Strategy—Depending on whether the parent individuals participate in the selection process or die beforehand, i.e., are removed from the further optimization process, the following variants of Evolution Strategy can be distinguished:

- **(μ + λ) – ES** (pronounced: mu plus lambda membered evolution strategy): With plus selection, the μ parents are added together with the λ offspring to the ballot box. Therefore, parents and offspring are included in the selection.
- **(μ, λ) – ES** (pronounced: mu comma lambda membered evolution strategy): With comma selection, the μ parents have a limited lifespan and are not added to the λ offspring in the ballot box. Therefore, the patents are not included in the selection.

Evolution window—Evolution, whether biological or artificial, can only take place within the evolution window (Fig. S2). For Evolution Strategy, this means that the individual changes caused by mutation must be neither too small nor too large. Therefore, the "mutation step size" must be chosen optimally. The closer you approach to the optimum, the smaller the steps you should take so as to avoid missing or skipping the optimum. Under certain circumstances, the step size must be refined during an optimization run. This is called “mutative step size control”.

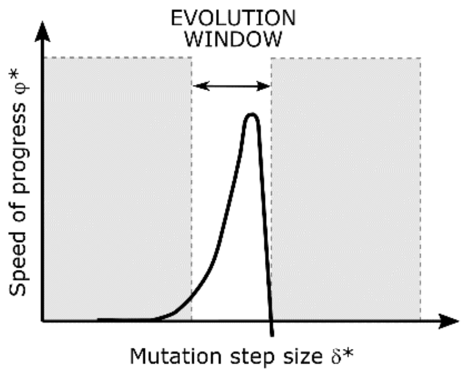

Figure S2: Only if the specific mutation step size δ is chosen optimally is there a realistic chance of finding the optimum within a reasonable time. If the mutation step size is too small, stagnation occurs because the specific speed of progress φ* is close to zero. If the mutation step size is too large, regression may occur because the speed of progress becomes negative.*

The Brachistochrone Problem

Is the shortest path also the fastest? Of all the curves, the straight line is undoubtedly the shortest geometrically. But does it also represent the one that is the shortest in time? This question can be answered either purely mathematically or with the help of Evolution Strategy, which, as in biological evolution, optimizes by trial and error.

A competition from 1696—The Swiss mathematician Johann Bernoulli was thinking about this question as early as 1696, when he posed the so-called brachistochrone problem originally in Latin to his fellow mathematicians: "Given two points A and B in a vertical plane, what is the curve traced out by a point acted on only by gravity, which starts at A and reaches B in the shortest time?'' [1]. Five solutions to Johann Bernoulli's problem were published by himself and other scientists in January and May 1697. The optimal trajectory is also known as the "fastest path" or "brachistochrone" (Greek: brachýs "short", chrónos "time").

The fastest and the shortest path—There are several ways to connect point A and point B, which are not at the same height and not directly below each other (Fig. S2.1). Both the shortest path (= a straight line) and the fastest path (= the brachistochrone curve) are optimization problems.

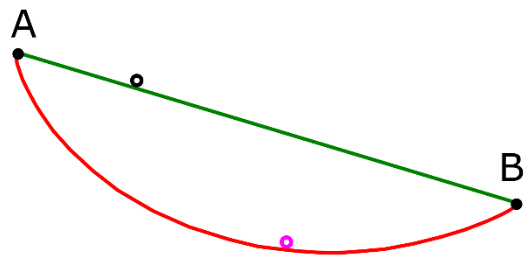

Figure S2.1: The points A and B can be connected by the shortest path (= a straight line, in green) and the fastest path (= the brachistochrone curve, in red). The black and magenta marbles start together at A but have traveled different distances by B.

The brachistochrone curve—Johann Bernoulli himself and some of his colleagues discovered that the brachistochrone has the shape of a cycloid. Such a cycloid is obtained by rolling a circle on a straight line and observing a fixed point on the edge of the circle (Fig. S2.2).

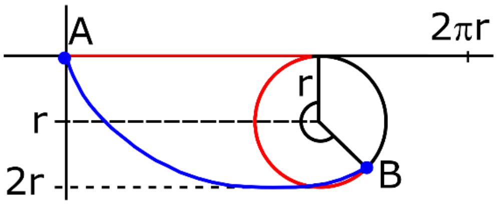

Figure S2.2: A cycloid is created by a rolling circle. If a *circle with a radius r rolls along a straight line, a fixed point on the edge of the circle describes a cycloid (blue) with a period of $2\pi r$ (= circumference).*

For all points on the cycloid, a marble starting at A will reach its destination fastest if it rolls along the cycloid. The really amazing thing is that the cycloid is the shortest path, even if A and B are so far apart that the marble has to roll slightly uphill!

Construction of the brachistochrone between given points—Given two points A and B to which you want to find the fastest path, you know that it has the shape of a cycloid, but to draw the corresponding cycloid segment, you need to know the radius of the corresponding circle. The radius can be easily determined by following the steps below:

1. The two given points A and B are connected by the line AB (Fig. S2.3).
2. Describe an arbitrary cycloid under the horizontal line through A, which only has to start at A. This cycloid will intersect the line AB at point R and the horizontal line at point S (Fig. S2.3).

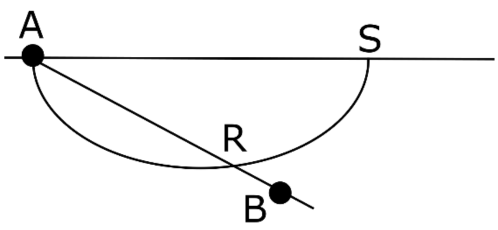

Figure S2.3: Construction steps 1 and 2 include the description of an arbitrary cycloid that intersects the line AB at point R.

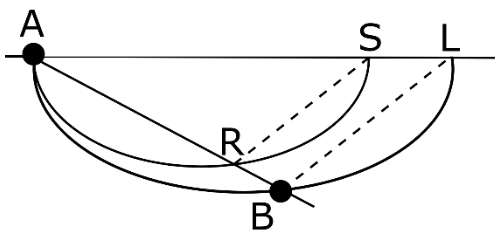

Figure S2.4: Construction step 3 to find the fastest path between A and B by centric stretching so that point R is mapped onto the point B.

3. The triangle ARS is now stretched by centric stretching so that the point R is mapped onto the point B. Since the image lines are always parallel to the original image line during centric stretching, the image line to RS can easily be drawn. It intersects the horizontal line at point L (Fig. S2.4).
4. With the 1st theorem of rays and the periodicity of the cycloids, it now follows that:

$$\frac{AB}{AR} = \frac{AL}{AS} = \frac{2\pi \cdot r_{ABL}}{2\pi \cdot r_{ARS}} = \frac{r_{ABL}}{r_{ARS}} \qquad (1)$$

   In equation (1) r_{ARS} is the circle radius of the given cycloid, and r_{ABL} is the circle radius of the cycloid being sought.

5. Solving the formula for r_{ABL} gives: $r_{ABL} = \frac{AB}{AR} \cdot r_{ARS}$ (2)
6. All the variables needed to calculate r_{ABL} are known, so the cycloid connecting points A and B can now be constructed easily.

[1] Bernoulli, J. Problema novum ad cujus solutionem Mathematici invitantur. *Acta Eruditorum* **1696**, 18.

Various Marble Track Shapes

Have you ever thought of an application in engineering when it would be better to use a brachistochrone curve instead of other path types? For an emergency slide, for example, we have to take the fastest route, which is the brachistochrone curve.

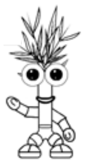	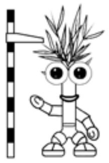	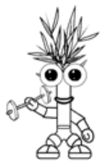	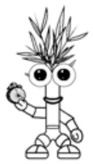
**Working type:** in teams or individually	**Age:** students older than 15 years	**Degree of difficulty:** Medium	**Duration:** 90 minutes

Introduction to the software "EvoBrach"—If you want to take a closer look at the brachistochrone problem, you can perform digital experiments simulating various marble track shapes by using the EvoBrach software. EvoBrach was developed to demonstrate the effectiveness of the Evolution Strategy in a complex optimization problem. The problem is to find the fastest path on which a marble rolls with negligible friction from start to end. For comparison, the running times obtained on a straight line and a parabola are also shown. EvoBrach uses a (1,10)–ES with covariance matrix adjustment to find the fastest path, which is a brachistochrone curve. This variant of Evolution Strategy continues until the termination criterion is met: if there is no change in the eighth decimal place of the running time in seconds for more than ten generations, then the optimization stops.

User interface and operating elements of EvoBrach

- Download the EvoBrach.zip file provided in Supplementary Materials File S4. Extract the files. Note that all files contained in the zip file must be located in one directory. EvoBrach.exe then only needs to be opened with a double click.
- At the start of the optimization with EvoBrach (Figure S3.1), the user can select the number of support points (= Stützstellenzahl) that divide the horizontal distance between the starting point and the end point into equidistant sections, and the diagram type (= Diagrammtyp) such as line, spline, line area, and spline area. If desired, the diagram types can also be displayed in 3D (tick the box of 3D-Darstellung).

- In addition, the user can also change the track geometry (= Bahngeometrie). Since the software has been developed in German, the numbers must be entered with decimal commas.
- Independently of the number of support points, a straight line (yellow) and a parabola (green) appear after the start button is pressed; both are fixed and serve as comparison models during the optimization.

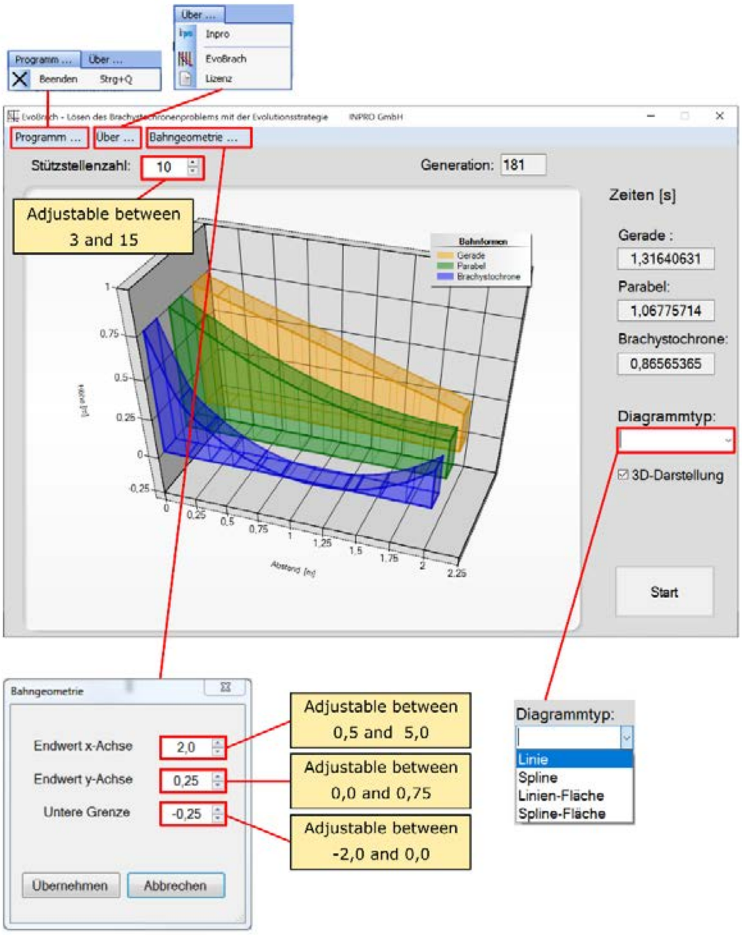

Figure S3.1: User interface and operating elements of EvoBrach

Performing the experiment

1. Open EvoBrach.exe with a double click.
2. Select the type of diagram.
3. Select whether the diagram should be displayed in3D.
4. Select the number of support points (between 3 and 15). Note the number in the table.
5. Press the “Start” button and observe the slow evolution of the brachistochrone.

6. Note the running times of the marble for the straight line, the parabola, and the brachistochrone curve in the table.
7. Repeat the simulation 3 times with different numbers of support points. Note the number of generations required for the optimization with the Evolution Strategy and the running times of the marble on the brachistochrone curve.
8. Complete the running times for the straight line and the parabola.

Task 1: Perform nine digital experiments with EvoBrach. Note the number of generations required to perform the optimization with the Evolution Strategy and the running times of the marble on the brachistochrone. Complete the running times for the straight line and the parabola.

**experiment #**	**number of support points**	**number of generations**	**running time of the brachistochrone curve [s]**
1			
2			
3			
4			
5			
6			
7			
8			
9			
			**straight line [s]:**
			**parabola [s]:**

Various Marble Track Shapes

Task 1: Perform nine digital experiments with EvoBrach. Note the number of generations required to perform the optimization with the Evolution Strategy and the running times of the marble on the brachistochrone. Complete the running times for the straight line and the parabola.

**experiment #**	**number of support points**	**number of generations**	**running time of the brachistochrone curve [s]**
1			
2			
3			
4			
5			
6			
7			
8			
9			
			**straight line [s]:**
			**parabola [s]:**

Various Marble Track Shapes

Task 2: Consider why, for the same number of support points, it takes a different number of generations to produce the optimized shape in terms of the brachistochrone.

____

____

____

____

____

____

____

____

Task 3: Explain why the brachistochrone curve sometimes looks highly irregular during optimization with the Evolution Strategy.

____

____

____

____

____

____

Various Marble Track Shapes

Task 1: Perform nine digital experiments with EvoBrach. Note the number of generations required to perform the optimization with the Evolution Strategy and the running times of the marble on the brachistochrone. Complete the running times for the straight line and the parabola (individual solution).

**experiment #**	**number of support points**	**number of generations**	**running time of the brachistochrone curve [s]**
1	15	312	0.86259096
2	15	292	0.86259096
3	15	337	0.86259098
4	3	59	0.88651092
5	3	60	0.88651092
6	3	53	0.88651092
7	10	175	0.86565364
8	10	151	0.86565365
9	10	154	0.86565365
			**straight line [s]:** 1.31640631
			**parabola [s]:** 1.0657159

Various Marble Track Shapes

Task 2: Consider why, for the same number of support points, it takes a different number of generations to produce the optimized shape in terms of the brachistochrone.

The marble track is optimized using the Evolution Strategy, namely the (1,10)–ES with covariance matrix adjustment. The offspring are generated by randomly changing the height of each support point. The offspring with the minimum running time becomes the parent of the next generation. The optimization is terminated according to a defined rule, namely if the eighth digit has not changed ten times in a row.

Task 3: Explain why the brachistochrone curve sometimes looks highly irregular during optimization with the Evolution Strategy.

On the basis of random processes (height of each individual support point), the proposed solutions for the curve are varied until the optimal solution is found for the brachistochrone curve. As the optimization proceeds, curves are created that randomly move up or down at each support point. The curve appears to "wriggle", which is an expression of the random process of Evolution Strategy, until the optimized brachistochrone curve is found.
