# Supplementary material for: The Basics of Evolution Strategies: The Implementation of the Biomimetic Optimization Method in Educational Modules
Source: Biomimetics (Basel). 2024 Jul 18;9(7):439. doi: 10.3390/biomimetics9070439 (PMC11274816; doi:10.3390/biomimetics9070439)
Supplement: Supplementary file 1 [file biomimetics-09-00439-s001.zip › S5_Statistik_ES.pdf]

google scholar search 08.05.2024
Search: "Evolution strategy" OR "Evolution strategies"
no patents
no citations

Year	number of publications
2023	6,610
2022	6,120
2021	5,170
2020	4,900
2019	4,710
2018	4,260
2017	3,770
2016	3,670
2015	3,600
2014	3,530
2013	3,450
2012	3,220
2011	2,990
2010	2,910
2009	2,770
2008	2,370
2007	2,310
2006	1,940
2005	1,730
2004	1,460
2003	1,270
2002	1,150
2001	1,010
**2000**	**771**
1999	821
1998	621
1997	527
1996	436
1995	322
1994	304
1993	188
**1992**	**94**
1991	74
1990	94
1989	50
1988	44
1987	26
1986	28
1985	25
1984	25
1983	20
1982	25

1981	17
1980	12
1979	10
1978	11
**1977**	**7**
1976	8
1975	4
1974	3
1973	1
1972	1
1971	0
**1970**	**20**
1969	1
1968	1
1967	0
1966	0
1965	3
1964	2
1963	2
1962	0
1961	0
1960	3
1959	3
1958	3
1957	0
1956	2
1955	0
Sum	79,529
