# Supplementary material for: The Basics of Evolution Strategies: The Implementation of the Biomimetic Optimization Method in Educational Modules
Source: Biomimetics (Basel). 2024 Jul 18;9(7):439. doi: 10.3390/biomimetics9070439 (PMC11274816; doi:10.3390/biomimetics9070439)
Supplement: Supplementary file 1 [file biomimetics-09-00439-s001.zip › S4_EvoBrach.zip!/S4_EvoBrach_Manual.pdf]

EvoBrach

Handbuch

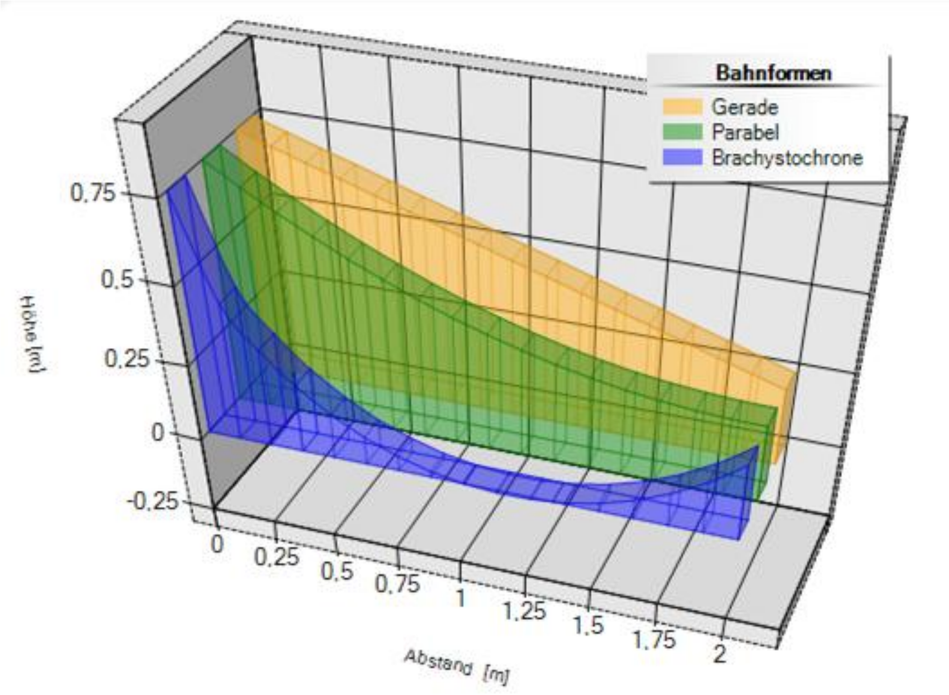

INPRO Innovationsgesellschaft für
fortgeschrittene Produktionssysteme
in der Automobilindustrie mbH

Dr.-Ing. Michael Herdy, Michael.Herdy@Inpro.de

August 2013

Inhaltsverzeichnis

1 BESCHREIBUNG 1

2 INSTALLATION 1

2.1 Benötigte Fremdsoftware 1

2.2 Installation von EvoBrach 1

3 OPTIMIERUNGSALGORITHMUS 1

4 FUNKTIONEN 2

4.1 Menü → Programm … → Beenden 2

4.2 Menü → Über … → Inpro 2

4.3 Menü → Über … → EvoBrach 2

4.4 Menü → Über … → Lizenz 2

4.5 Stützstellenzahl 2

4.6 Bahngeometrie → Endwert x-Achse 2

4.7 Bahngeometrie → Endwert y-Achse 2

4.8 Bahngeometrie → Untere Grenze 2

4.9 Start / Stop 2

4.10 Diagrammtyp 2

4.11 3D-Darstellung 2

5 ANZEIGE 3

5.1 Generationen 3

5.2 Zeiten [s] 3

6 HAFTUNGSAUSSCHLUSS 3

7 EvoBrach: Oberfläche und Bedienelemente 4

1 Beschreibung

Die Software **EvoBrach** dient zur Demonstration der Wirkungsweise der Evolutionsstrategie bei der Optimierung eines komplexen Optimierungsproblems. Gefragt ist nach der Bahnkurve, auf der eine Kugel bei vernachlässigbarer Reibung vom Start zum Ziel rollt. Zum Vergleich werden auch die Zeiten dargestellt, die auf einer Geraden bzw. einer Parabel erzielt werden. Die schnellste Bahn ist die so genannte Brachystochrone.

2 Installation

2.1 Benötigte Fremdsoftware

Zur Funktion von **EvoBrach** wird vorausgesetzt, dass sich das **Microsoft .NET Framework Version 3.5** auf Ihrem System befindet. Diese Software ist nicht im Lieferumfang von EvoBrach enthalten und kann über folgenden Link kostenfrei bezogen werden:
http://www.microsoft.com/downloads/details.aspx?familyid=d0e5dea7-ac26-4ad7-b68c-fe5076bba986&displaylang=de

Welche Softwarepakete auf Ihrem PC installiert sind, finden Sie unter: Start → Einstellungen → Systemsteuerung → Software. Zur Installation des .NET Frameworks benötigen Sie Administratorrechte auf dem Zielrechner.

2.2 Installation von EvoBrach

Für **EvoBrach** selbst ist keine Installation erforderlich; es müssen sich aber alle im zip-file enthaltenen Dateien in einem Verzeichnis befinden. EvoBrach.exe muss dann nur ausgeführt werden.

3 Optimierungsalgorithmus

Realisiert wurde eine (1, 10)-ES mit Anpassung der Kovarianzmatrix. Als Abbruchkriterium wird der Fortschritt geprüft; wenn mehr als zehn Generationen lang auf der achten Stelle nach dem Komma keine Änderung erfolgte, stoppt die Optimierung.

4 Funktionen

Eine Übersicht über die Funktionen gibt die Abbildung auf Seite 5.

4.1 Menü → Programm … → Beenden

Das Programm wird beendet. Alternativ kann dazu mit der linken Maustaste das Kreuz in der rechten oberen Ecke des Programm-Fensters angewählt werden.

4.2 Menü → Über … → Inpro

Es wird die Internetseite von Inpro aufgerufen.

4.3 Menü → Über … → EvoBrach

Es erscheint eine kurze Programminformation.

4.4 Menü → Über … → Lizenz

Es erscheint eine Information zum Haftungssausschluss (siehe auch Abschnitt 6)

4.5 Stützstellenzahl

Es können Stützstellenzahlen zwischen 3 und 15 vorgegeben werden. Der horizontale Abstand der Stützstellen ist immer äquidistant und wird während der Optimierung nicht verändert. Mit Änderung der Stützstellenzahl werden jeweils auch die Gerade und die Parabel sowie die Ausgangslage der zu optimierenden Kurve neu gezeichnet und die Abrollzeit für diese Stützstellenzahl berechnet. Zu Beginn liegen die zu optimierende Kurve und die Gerade übereinander.

4.6 Bahngeometrie → Endwert x-Achse

Für den Endwert der x-Achse können Werte zwischen 0,5 und 5,0 eingestellt werden.

4.7 Bahngeometrie → Endwert y-Achse

Für den Endwert der y-Achse können Werte zwischen 0,0 und 0,75 eingestellt werden.

4.8 Bahngeometrie → Untere Grenze

Die untere Grenze bestimmt den tiefsten möglichen Wert für die Stützstellen, den die Evolutionsstrategie einstellen kann. Dieser kann zwischen 0,0 und -2,0 vorgegeben werden.

4.9 Start / Stop

Die Optimierung wird gestartet bzw. beendet.

4.10 Diagrammtyp

Für die Darstellung der Kurven kann aus den Darstellungsmodi „Linie“, „Spline“, „Linien-Fläche“ und „Spline-Fläche“ gewählt werden.

4.11 3D-Darstellung

Es erfolgt eine Umschaltung zwischen 2D- und 3D-Darstellung der Kurven.

5 Anzeige

5.1 Generationen

Die Zahl der Generationen seit Start der Optimierung wird angezeigt.

5.2 Zeiten [s]

Die Zeit vom Start bis zum Erreichen des Zielpunktes in [s] wird angezeigt.

6 Haftungsausschluss

Die Software und Ihre Dokumentation wird "wie sie ist" und ohne jede Gewährleistung für Funktion, Korrektheit oder Fehlerfreiheit zur Verfügung gestellt.

Für jedweden direkten oder indirekten Schaden - insbesondere Schaden an anderer Software, Schaden an Hardware, Schaden durch Nutzungsausfall und Schaden durch Funktionsuntüchtigkeit der Software, kann der Autor nicht haftbar gemacht werden.

Ausschließlich der Benutzer haftet für die Folgen der Benutzung dieser Software.

Diese Software wurde mit größter Sorgfalt entwickelt, jedoch können Fehler niemals ausgeschlossen werden. Es kann daher keine Gewähr für die Sicherheit Ihrer Daten übernommen werden.

7 **EvoBrach:** Oberfläche und Bedienelemente

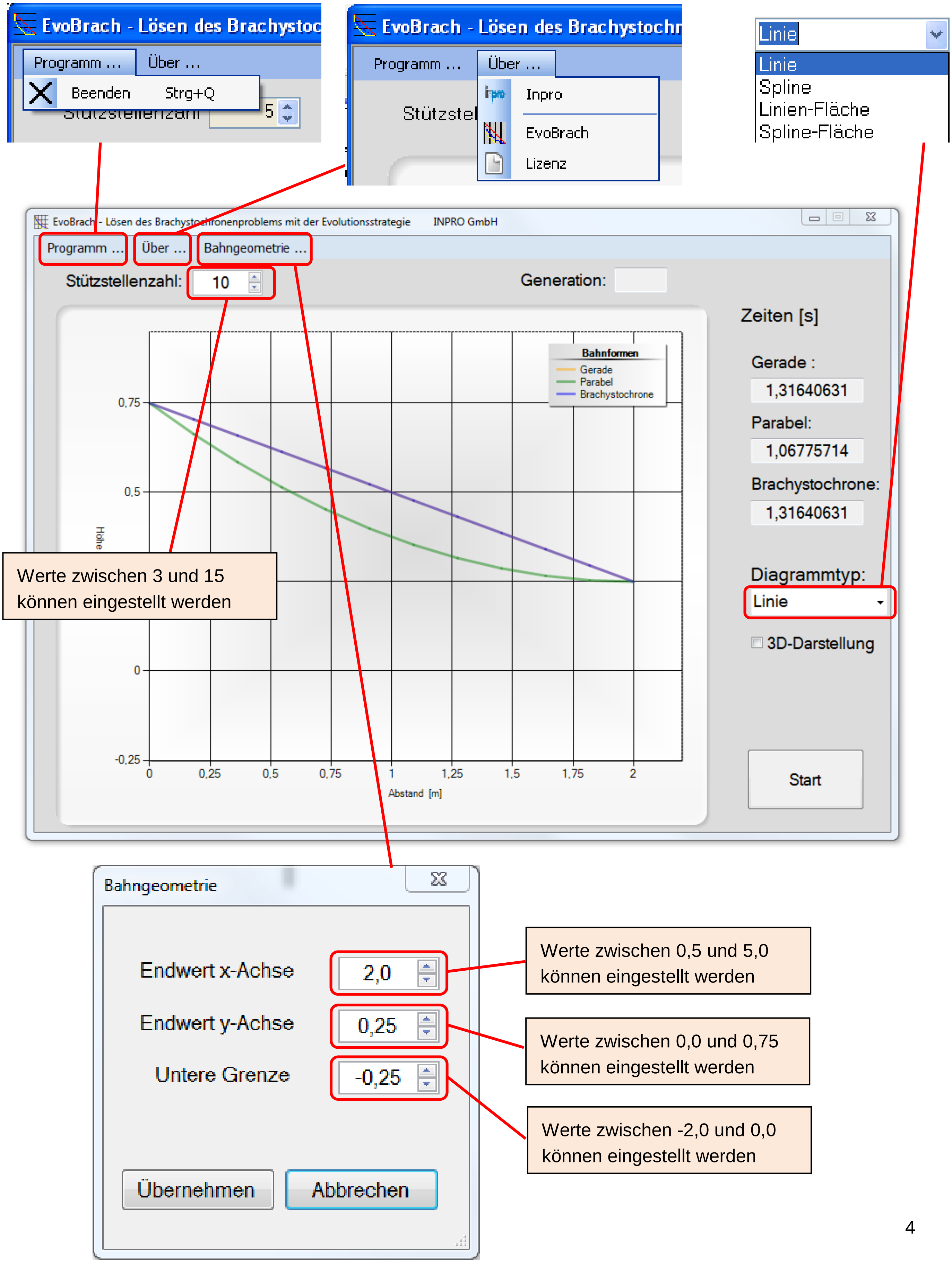

Dieses Projekt wurde mit Mitteln des Bundesministerium für Bildung und Forschung (FKZ 01RB0708B) im Rahmen der Förderrichtlinie "BIONA - Bionische Innovationen für nachhaltige Produkte und Technologien“ gefördert.
